# Single-cell multi-ome regression models identify functional and disease-associated enhancers and enable chromatin potential analysis

**DOI:** 10.1038/s41588-024-01689-8

**Published:** 2024-03-21

**Authors:** Sneha Mitra, Rohan Malik, Wilfred Wong, Afsana Rahman, Alexander J. Hartemink, Yuri Pritykin, Kushal K. Dey, Christina S. Leslie

**Affiliations:** 1 Computational and Systems Biology Program, Memorial Sloan Kettering Cancer Center, New York City, NY USA; 2 Rye Country Day School, Rye, NY USA; 3 Tri-Institutional Training Program in Computational Biology and Medicine, New York City, NY USA; 4 Hunter College, City University of New York, New York City, NY USA; 5 Department of Computer Science, Duke University, Durham, NC USA; 6 Program in Computational Biology and Bioinformatics, Duke University, Durham, NC USA; 7 Center for Genomic and Computational Biology, Duke University, Durham, NC USA; 8 Department of Computer Science, Princeton University, Princeton, NJ USA; 9 Lewis-Sigler Institute for Integrative Genomics, Princeton University, Princeton, NJ USA

**Keywords:** Software, Population genetics, Epigenetics

## Abstract

We present a gene-level regulatory model, single-cell ATAC + RNA linking (SCARlink), which predicts single-cell gene expression and links enhancers to target genes using multi-ome (scRNA-seq and scATAC–seq co-assay) sequencing data. The approach uses regularized Poisson regression on tile-level accessibility data to jointly model all regulatory effects at a gene locus, avoiding the limitations of pairwise gene–peak correlations and dependence on peak calling. SCARlink outperformed existing gene scoring methods for imputing gene expression from chromatin accessibility across high-coverage multi-ome datasets while giving comparable to improved performance on low-coverage datasets. Shapley value analysis on trained models identified cell-type-specific gene enhancers that are validated by promoter capture Hi-C and are 11× to 15× and 5× to 12× enriched in fine-mapped eQTLs and fine-mapped genome-wide association study (GWAS) variants, respectively. We further show that SCARlink-predicted and observed gene expression vectors provide a robust way to compute a chromatin potential vector field to enable developmental trajectory analysis.

## Abstract (web summary)

Single-cell ATAC + RNA linking (SCARlink) predicts gene expression by jointly modeling local tiled chromatin accessibility using regularized Poisson regression on multi-ome data. SCARlink predictions can be used to identify cell-type-specific enhancers and perform chromatin potential analysis.

## Main

Multi-ome single-cell sequencing of chromatin accessibility and gene expression—where both scATAC–seq and scRNA-seq are applied to the same individual cells—has paved the way for computational methods that attempt to link enhancers to genes^1,2^, infer gene regulatory networks^3–5^ and resolve developmental trajectories based on the concept of chromatin potential, which proposes that accessibility at a locus precedes gene expression during differentiation^1^. At the most elementary level, several approaches exploit joint measurements of ATAC and RNA in single cells to identify pairwise correlations between individual accessible regions—defined as peaks or domains of open chromatin (DORCs)—and gene expression levels for enhancer–gene linking^1,6^. For example, a recent approach uses Poisson regression to test for pairwise correlation between peak accessibility and gene expression while also modeling batch or cell-specific covariates, with the goal of linking noncoding genetic variants that reside in such peaks to target genes^2^. Meanwhile, standard scATAC–seq analysis methods use simple scoring schemes to transform the data into a scRNA-like readout, analogous to gene expression, based on aggregating chromatin accessibility near a gene promoter or across a genic locus, comprising the gene body and a fixed window around it, to obtain an imputed gene expression value. These imputation scores enable joint embedding of independently collected scATAC–seq and scRNA-seq data or transfer of cell-type cluster labels between the two^7^.

**Fig. 1:**
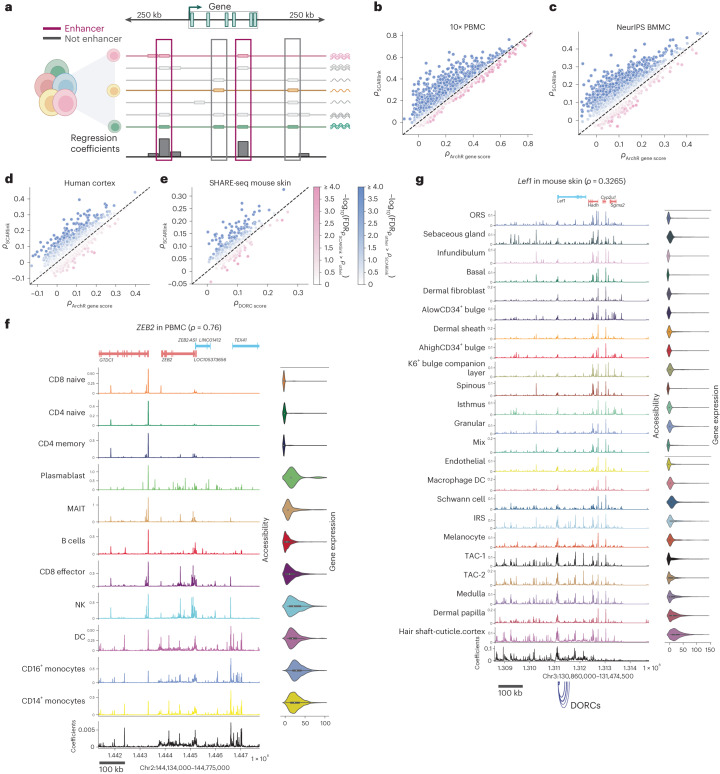
SCARlink accurately predicts single-cell gene expression from chromatin accessibility. **a**, The model takes as input single-cell ATAC–seq counts at a genic locus, aggregated over 500 bp tiles spanning 250 kb upstream/downstream and including the gene body, and uses regularized Poisson regression to predict the gene’s single-cell expression; both the scATAC–seq and scRNA-seq readouts are obtained from multi-omic sequencing. The learned regression coefficients indicate the importance of each tile for predicting gene expression. **b**–**e**, Scatterplots showing Spearman correlation of predicted and observed gene expression for each gene using SCARlink versus Spearman correlations using existing methods. Comparisons are performed against ArchR gene score predictions on 10× PBMC (1,250 genes; **b**), BMMC^8^ (1,655 genes; **c**) and developing human cortex^9^ (1,201 genes; **d**); and against DORC gene score predictions (**e**) on the mouse skin^1^ dataset (380 genes). **f**, Example model output for *ZEB2* from PBMC multi-ome data (*n* = 9,460 cells; Supplementary Table 1), showing regression coefficients at bottom and aggregated scATAC- (left) and scRNA-measured expression (right) by cell type. **g**, Example model output and comparison with annotated DORCs (shown using blue arcs below the coefficient panel) for *Lef1* from mouse skin SHARE-seq data (*n* = 33,314 cells; Supplementary Table 1). *ρ* indicates the Spearman correlation between predicted and observed gene expression. The gene expression depicted using violin plots in **f** and **g** are normalized to counts per 10,000. The boxplots inside the violin plots for gene expression in **f** and **g** are centered on the median, bounded by the quartiles, with the whiskers depicting the remaining distribution. This image is created with BioRender.com.

Motivated by these ideas, we propose single-cell ATAC + RNA linking (SCARlink), a gene-level predictive model for single-cell/single-nucleus multi-ome data that predicts the expression of a gene from the accessibility of its genomic context in single cells (Fig. 1a). Unlike pairwise correlation approaches that assess individual peak–gene links independently, our model captures the fact that elements both within the genic locus (for example, intronic enhancers) and distal elements in flanking regions (±250 kb by default) all jointly regulate the expression of the gene. We train the model using regularized Poisson regression on tile-level data to facilitate integration with standard preprocessing pipelines like ArchR^6^ and to avoid summarizing data as a peak atlas, which not only requires additional steps for peak calling over clusters but may miss events in rarer cell types. The regression coefficients across the genomic context can then be interpreted as identifying locations of putative candidate functional enhancers across the single-cell dataset. Moreover, we can use Shapley values, a well-known feature attribution method, to identify cell-type-specific enhancers, that is, genomic tiles that are important for predicting expression across cells from a given cluster or annotation. Therefore, although SCARlink is formulated as a gene expression prediction problem, we can use the learned model parameters to infer enhancer–gene links in a cell-type-specific manner. Below, we show that our model outperforms existing methods for predicting single-cell gene expression from accessibility and correctly identifies cell-type-specific enhancers, as validated by promoter capture Hi-C (PCHi-C). We further show that the regulatory regions determined using Shapley values from our modeling enrich for fine-mapped noncoding genome-wide association study (GWAS) and expression quantitative trait loci (eQTL) variants. Finally, we demonstrate that using gene-level models for a set of developmentally regulated genes yields a robust implementation of the chromatin potential trajectory inference method.

## Results

### SCARlink accurately predicts gene expression and identifies putative enhancers

SCARlink uses a regularized Poisson regression model on single cells to predict gene expression from chromatin accessibility. The chromatin accessibility is used as input in the form of nonoverlapping 500 bp tiles spanning a region from 250 kb upstream to 250 kb downstream of the gene body by default (Fig. 1a). This genomic context is large enough to capture distal intergenic as well as intronic enhancers for most genes but can be extended or shortened as preferred. Because SCARlink is a gene-level model and genes are of variable length, the number of input tiles is different for every gene. For example, for genes *CCR7* and *ZEB2*, 11,703 bp and 140,502 bp long, the number of input tiles was 1,024 and 1,282, respectively. We also constrain the model to learn positive regulatory elements by forcing the regression coefficients to be non-negative. While this is a limitation for identifying repressive regulatory elements, we found the regression coefficients to be more interpretable when we focused on enhancers.

**Extended Data Fig. 1:**
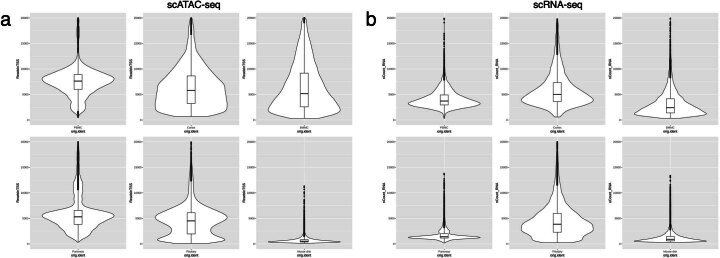
Comparison of sparsity of scATAC-seq and scRNA-seq. **a**. Distribution of fragments near TSS (ReadsInTSS) from scATAC-seq for all six data sets. **b**. Distribution of UMI counts from scRNA-seq for all six data sets. The boxplots inside the violin plots are centered on the median, bounded by the quartiles, with the whiskers depicting the remaining distribution.

We applied SCARlink to multi-omic datasets of different levels of sparsity. Datasets with lower levels of sparsity include peripheral blood mononuclear cells (PBMCs) from 10X Genomics (mean UMI counts: 4,172, mean reads in TSS (transcription start site): 7,682), bone marrow mononuclear cells (BMMCs^8^; mean UMI counts: 3,278, mean reads in TSS: 7,134) and developing human cortex^9^ (mean UMI counts: 6,344, mean reads in TSS: 6,874); datasets with higher sparsity are mouse skin^1^ (mean UMI counts: 1,244, mean reads in TSS: 707), pancreas^10,11^ (mean UMI counts: 6,445, mean reads in TSS: 1,830) and pituitary gland^12^ (mean UMI counts: 4,786, mean reads in TSS: 4,615; Extended Data Fig. 1). We ran the model on a subset of the top 5,000 most variable genes for each dataset, filtered based on the sparsity of the gene expression vector (Methods). After filtering, we obtained 1,250 genes for PBMC, 1,655 genes for BMMC, 393 genes for mouse skin, 1,201 genes for the developing human cortex, 784 genes for the pancreas and 1,221 genes for the pituitary gland (Supplementary Table 1). For each gene-level model, we used Spearman correlation to compare the predicted gene expression to observed gene expression on held-out cells.

We compared SCARlink against other available methods to predict single-cell gene expression from chromatin accessibility. One such method is the ArchR gene score, which aggregates accessibility across the gene body and flanking regions using an exponentially decaying function to downweight accessibility farther away from the gene. SCARlink significantly outperformed the ArchR gene score across all high-coverage datasets based on correlation with ground truth on held-out cells (one-sided signed-rank test over genes, *P* < 8.35 × 10^−114^on PBMC, *P* < 3.24 × 10^−200^on BMMC and *P* < 1.15 × 10^−61^on developing human cortex). We also found that SCARlink produced significantly higher correlations for a large fraction of individual genes in higher coverage datasets (57.0% of genes in PBMC, 56.8% of genes in BMMC and 24.4% of genes in the developing cortex, at false discovery rate (FDR) < 0.05) as assessed by pairwise significance of correlation (Methods; Fig. 1b–d).

**Extended Data Fig. 2:**
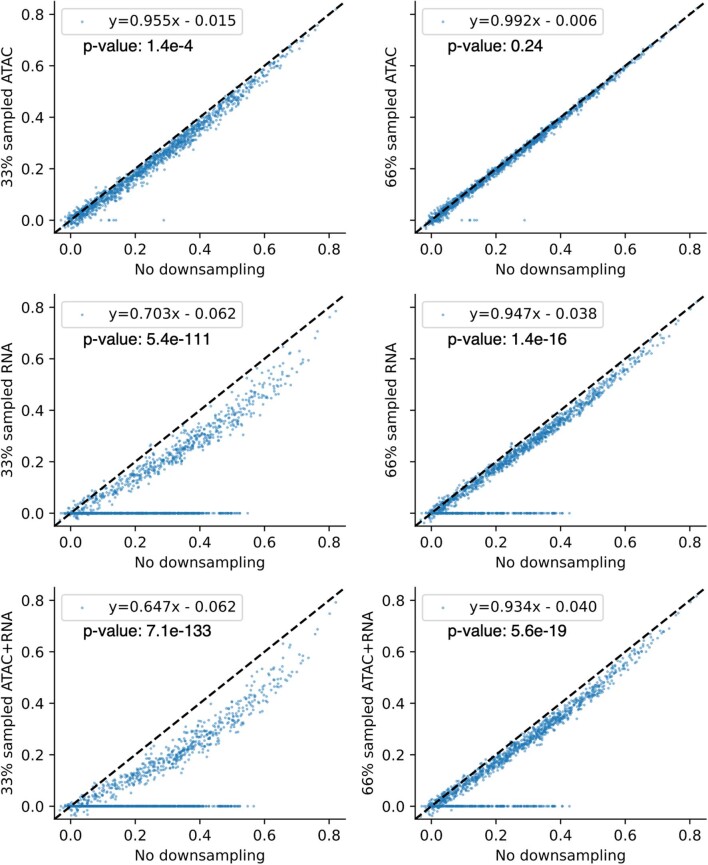
Count down-sampling of PBMC multi-ome. Comparison Spearman correlations of SCARlink predictions on PBMC multi-ome with and without count downsampling of scATAC-seq (top row), scRNA-seq (second row), or both (bottom row). Linear least-squares regression was performed to determine slope and intercept. A two-sided Mann-Whitney U test was used to assess significance.

**Extended Data Fig. 3:**
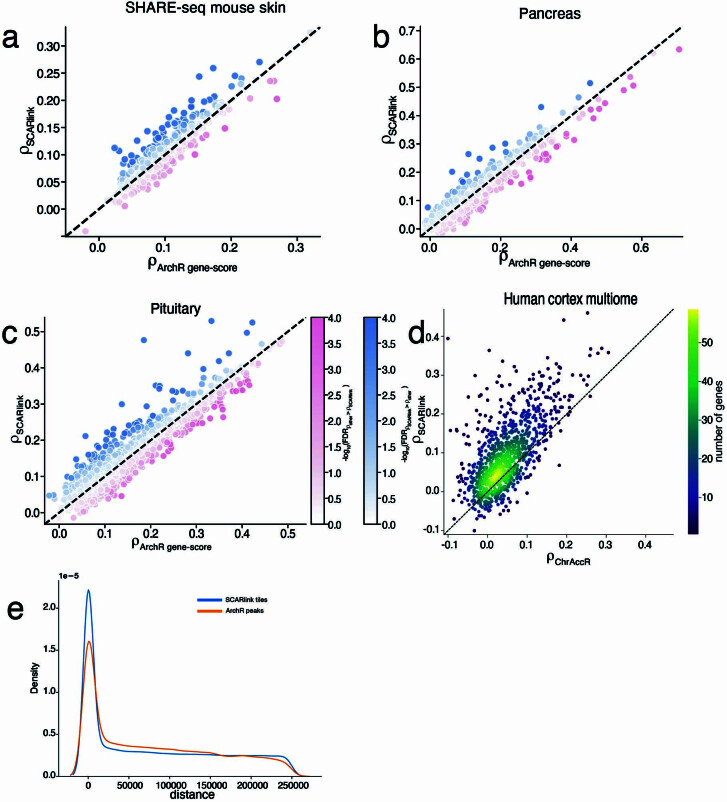
SCARlink prediction of gene expression compared to existing methods. **a**–**c**. Comparison of Spearman correlation of prediction of gene expression between SCARlink and ArchR gene score in (**a**) mouse skin^1^, (**b**) pancreas^10,11^, and (**c**) pituitary multi-ome^12^. For each of the scatterplots in **a**–**c**, a significance score is computed between the Spearman correlations of SCARlink and ArchR gene score. The dots are colored based on the p-values (one-sided). **d**. Comparison of Spearman correlation of prediction of gene expression between SCARlink and ChrAccR in developing human cortex multi-ome. Each dot is a single gene. We use the Spearman correlations pre-computed using ChrAccR scores for each gene as previously reported^9^. Hence, no statistical tests were performed. Here the dots are colored based on the density of genes. **e**. Smoothed distribution plot depicting the number regulatory regions predicted within SCARlink tiles (blue) and ArchR paired peak-gene links (orange) is highest within the gene body(dist in basepairs=0) and decreases on moving further away from the gene body.

We determined through count downsampling of PBMC that the sparsity of scATAC–seq and/or scRNA-seq substantially affects model performance (Extended Data Fig. 2). Thus, for sparser datasets (Extended Data Fig. 1), SCARlink performed comparably to the ArchR gene score on pancreas and pituitary (one-sided signed-rank test is not significant in either direction) while outperforming it on mouse skin (*P* < 3.7 × 10^−09^, one-sided signed-rank test), albeit with fewer genes showing significantly better correlation (Extended Data Fig. 3a–c and Supplementary Table 1). In the human cortex multi-ome data, SCARlink outperformed another method of gene score prediction called ChrAccR that aggregates the accessibility in peaks near the TSS (*P* < 1.3 × 10^−93^, one-sided signed-rank test; Extended Data Fig. 3d).

**Extended Data Fig. 4:**
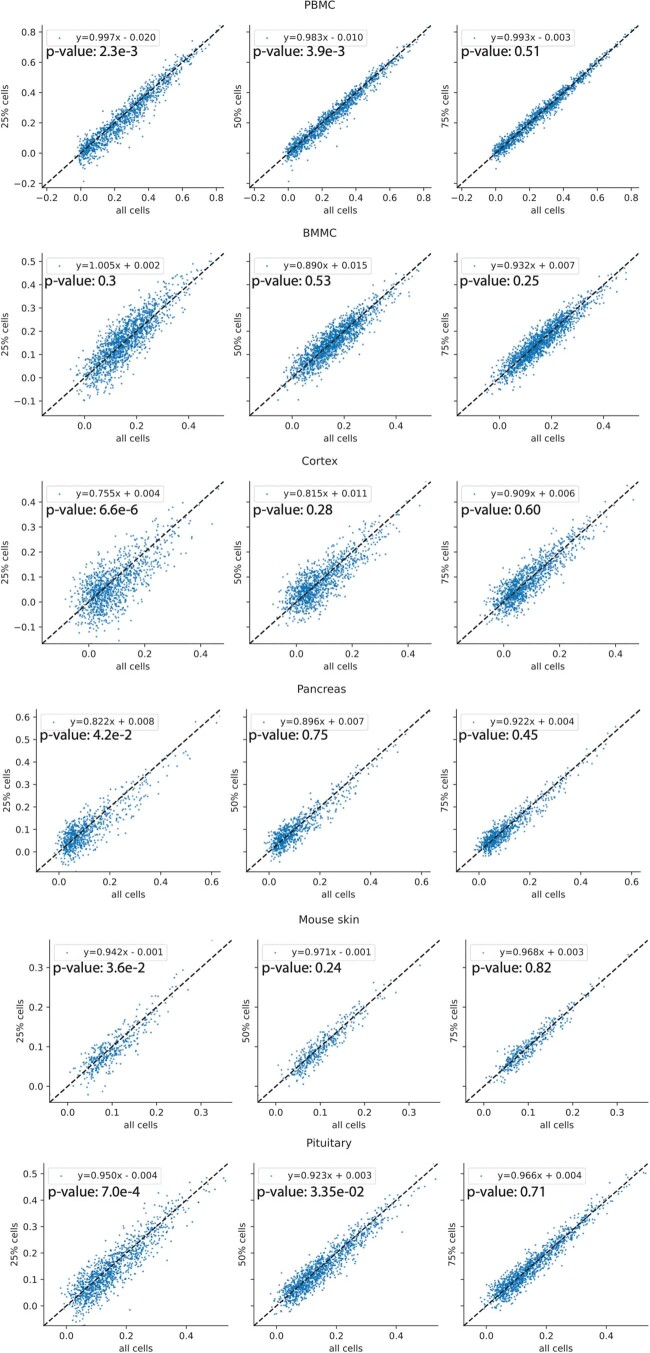
SCARlink predictions are robust to down-sampling number of cells. SCARlink was run with 25% (first column), 50% (second column), and 75% (third column) of the original number of cells. Spearman correlations for these down-sampled models on y-axis are compared to the correlations of predictions obtained using the original model (x-axis). Linear least-squares regression was performed to determine slope and intercept. A two-sided Mann-Whitney U test was used to assess significance.

DORC scores are computed by aggregating accessibility in peaks lying within 50 kb and 500 kb of the TSS that individually correlate with gene expression^1^. We found that our model yields predictions that are more correlated with expression than DORC scores in mouse skin (*P* < 3.1 × 10^−18^, one-sided signed-rank test; significantly better performance on 38.4% of genes; Fig. 1e), potentially because SCARlink is modeling the impact of chromatin accessibility across all tiles at once. In addition, we found that SCARlink predictions are robust to downsampling of the number of cells, yielding comparable predictions with at least 50% of the total number of cells across most datasets (Extended Data Fig. 4).

As an example to study the linkage between chromatin accessibility and gene expression, we used SCARlink to model the regulation of *ZEB2* in the PBMC dataset (Fig. 1f). The learned regression coefficients across all the tiles (Fig. 1f, bottom) identify candidate functional enhancers across the genomic locus for *ZEB2*. Note that while SCARlink does not use cell type or cluster annotations as input, knowledge of clusters can be used to generate pseudobulk visualizations and thus interpret the regression coefficients. We also analyzed *Lef1* from mouse skin multi-omic SHARE-seq data and found distal regions where high regression coefficients indicate that accessibility is correlated with transcription but which are not annotated as DORCs (near chromosome (chr)3:130,900,000; Fig. 1g). This highlights the advantage of SCARlink in using accessibility across all tiles for the prediction of gene expression.

### Shapley analysis identifies cell-type-specific SCARlink enhancers

The regression coefficients generated using SCARlink indicate the overall importance of the accessibility in each tile for predicting gene expression across cells in the dataset. To quantify the contribution of each tile in the window for every cell type, we computed standardized average Shapley values per cell type (see Methods for computation of approximate Shapley scores under the SCARlink model). This allowed us to identify tiles as putative regulatory regions for the modeled gene in a particular cell type. We observed that predicted regulatory elements are most enriched within or in close proximity to the gene body (∼25 kb) and decrease in prevalence in distal regions (Extended Data Fig. 3e).

Because active enhancers are known to physically interact with promoters to enable transcription^13^, we hypothesized that SCARlink-predicted regulatory regions would be enriched for 3D interactions with the promoter of the modeled gene. PCHi-C is a chromosome conformation capture assay that identifies promoter-interacting genomic regions using a genome-wide promoter bait library. We, therefore, sought to validate SCARlink-predicted regulatory regions across a subset of PBMC cell types using available hematopoietic cell PCHi-C^14^. We identified PCHi-C interactions in relevant cell types using a generalized additive model (Methods) and compared them to SCARlink-identified regions in T cell subpopulations, monocytes and B cells in the PBMC multi-omic data.

**Fig. 2:**
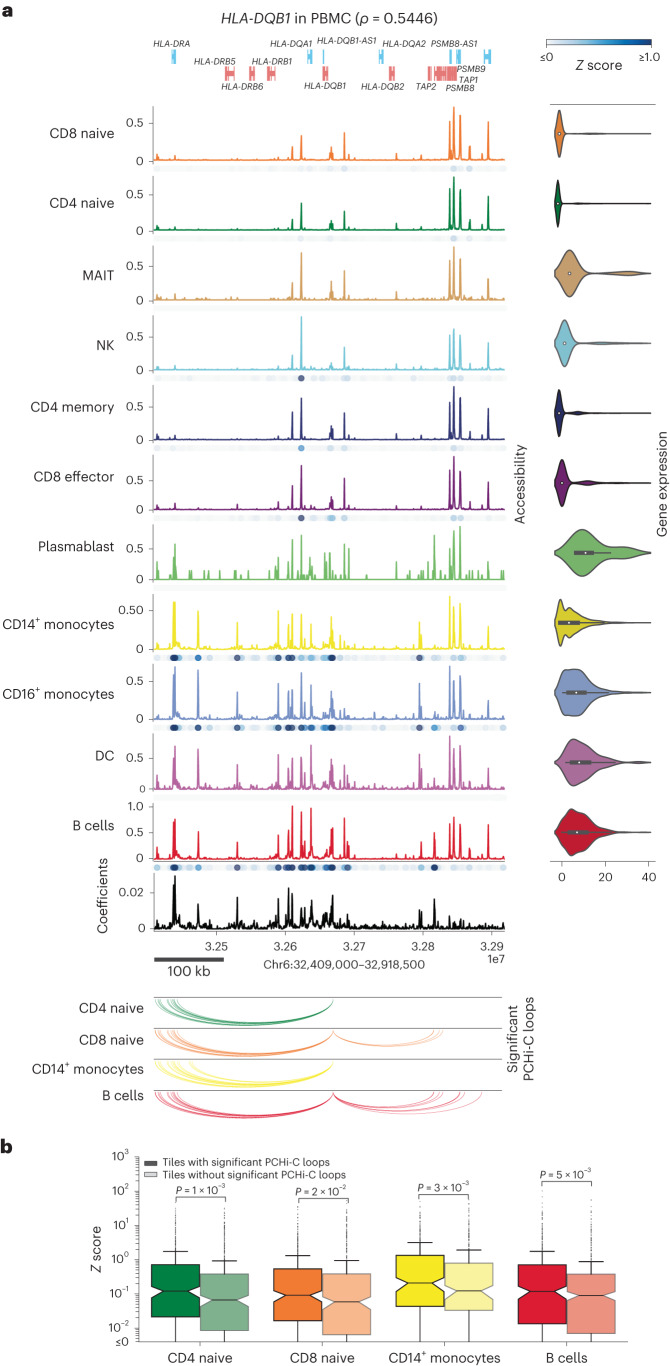
SCARlink coefficients enrich for promoter-linked chromatin interactions. **a**, SCARlink output of *HLA-DQB1* in PBMC multi-ome (*n* = 9,460 cells; Supplementary Table 1). Cell-type-specific standardized approximate Shapley scores (*z*scores) of the tiles are plotted as blue dots under the accessibility panel of every cell type. Arc plots of significant PCHi-C interactions^14^ (Methods; Supplementary Table 2) for *HLA-DQB1*of CD4 naive T, CD8 naive T, CD14^+^ monocytes and B cells are shown below the model output. The boxplots inside the violin plots for gene expression in **a** are centered on the median, bounded by the quartiles, and the whiskers depict the remaining distribution. **b**, Boxplots comparing feature scores of tiles with or without PCHi-C interactions (Supplementary Table 2) for highly expressed genes per cell type. Significance estimated using one-sided Mann–Whitney *U* test. The boxplots in **b** are centered on the median, bounded by the quartiles, with the whiskers extending up to values within 1.5× IQR, and the remaining points as outliers. IQR, interquartile range.

As one example, we compared our Shapley values to PCHi-C interactions for the gene *HLA-DQB1* (Fig. 2a). We found that PCHi-C interactions in distal tiles display higher Shapley values than noninteracting tiles, particularly for B cells, a cell type in which *HLA-DQB1* is highly expressed (Fig. 2a). We then compared the Shapley values of tiles with and without PCHi-C interactions for highly expressed genes in each cell type (Methods) and confirmed that Shapley values for interacting tiles are substantially higher than noninteracting tiles (Fig. 2b and Supplementary Table 2).

### Predicted enhancers are enriched for fine-mapped GWAS and eQTL variants

**Fig. 3:**
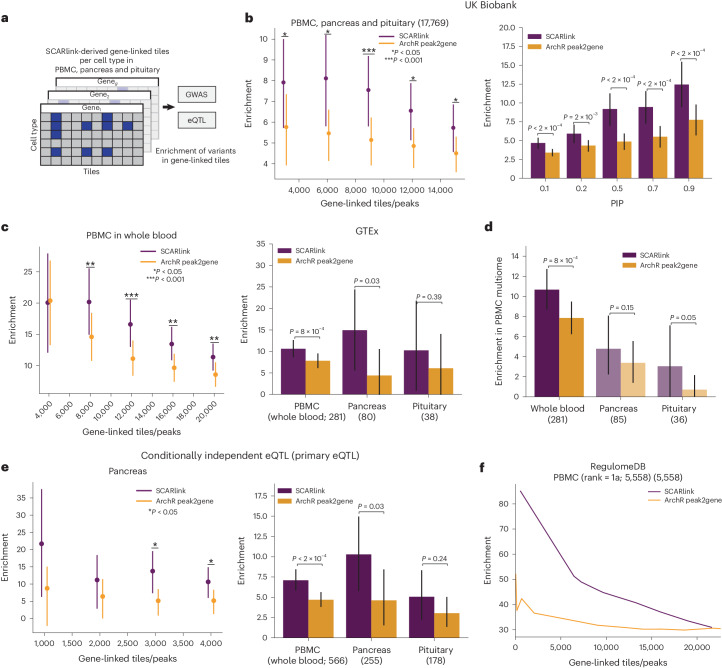
SCARlink-predicted gene-linked tiles enrich for causal variants. **a**, Schematic depicting the filtering of gene-linked tiles per cell type from SCARlink output of genes from PBMC, pancreas and pituitary multi-ome. These filtered gene-linked tiles are then checked for enrichment of causal variants from GWAS, eQTLs and other variant databases. **b**, Bootstrapped mean enrichment of 17,769 fine-mapped GWAS variants from UK Biobank (Supplementary Table 3) in the gene-linked SCARlink tiles (purple; Supplementary Table 4) and ArchR peak2gene peaks (yellow) as a function of the number of gene-linked tiles/peaks for PIP threshold of 0.2 (left). Comparison of enrichment at different PIP thresholds (right). The bars depicting a 95%CI of enrichment were obtained by bootstrapping traits. A total of 1,000 bootstrap iterations were used. *P < 0.05 and ***P < 0.001. **c**, Bootstrapped mean enrichment of 281 fine-mapped eQTLs from whole-blood GTEx in PBMC multi-ome (left). Comparison of enrichment in the matched GTEx tissue as the multi-ome datasets (right). The number of fine-mapped variants per tissue is mentioned in parenthesis. *P < 0.05 and ***P < 0.001. **d**, Comparison of bootstrapped mean enrichment of eQTLs from GTEx tissues (pituitary, pancreas and whole blood) in PBMC multi-ome. **e**, Bootstrapped mean enrichment of 255 primary independent eQTLs from the pancreas as a function of a number of gene-linked tiles/peaks (left). Enrichment of primary eQTLs in matched tissues in PBMC, pancreas and pituitary (right). *P < 0.05. The bars depicting a 95%CI of enrichment in **c**–**e** were obtained by bootstrapping genes. Two-sided bootstrapped *P* values are plotted in **b**–**e**. A total of 1,000 bootstrap iterations were used. **f**, Enrichment of 5,558 variants from RegulomeDB of rank = 1a in PBMC multi-ome. CI, confidence interval.

**Extended Data Fig. 5:**
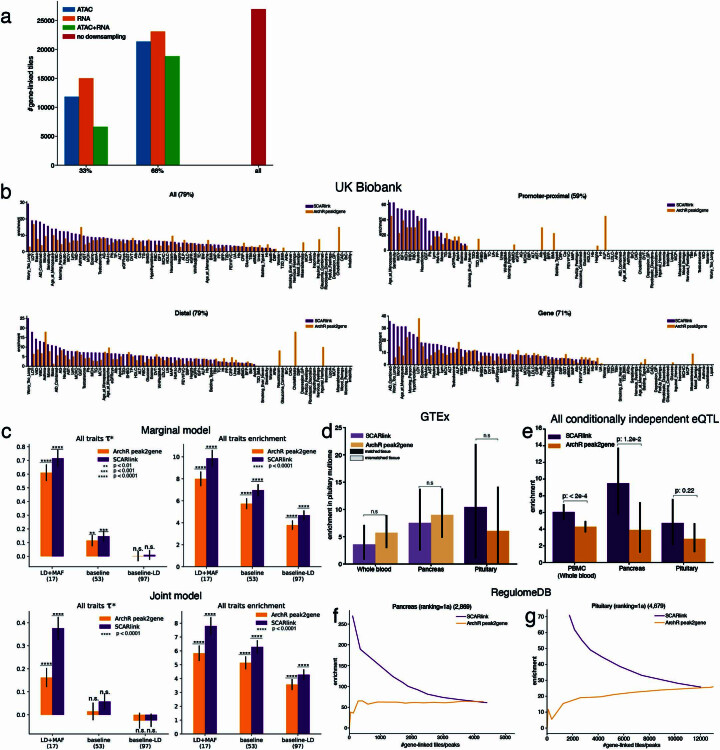
Comparison of variant enrichment in gene-linked tiles/peaks from SCARlink and ArchR peak2gene. **a**. Number of predicted gene-linked tiles in PBMC multi-ome upon down-sampling scATAC-seq, scRNA-seq, or both. Downsampling is performed either to 33% or 66% of original counts. **b**. Trait-specific enrichment of fine-mapped GWAS variants from UK Biobank for PIP > 0.2. Enrichment for a trait is 0 when no variants are found in predicted gene-linked tiles/peaks. Enrichment is plotted for different distance grouping. The percentage in the title of the plots indicate the number of traits for which SCARlink enrichment is greater than or equal to ArchR enrichment. **c**. S-LDSC meta-analyzed enrichment and τ* calculated using marginal heritability (top), and joint heritability (bottom) model for 82 traits for genes common to both SCARlink (mean annotation: 7e-3) and ArchR (mean annotation: 8e-3). Error bars indicate standard errors. All bars are significant unless otherwise specified. Two-sided p-values are estimated using meta-analysis (Supplementary Table 6). The *P* values are defined as **: p < 0.1; ***: p < 0.001; ****: p < 0.0001. **d**. Bootstrapped mean enrichment of pituitary multi-ome for GTEx pituitary, and other GTEx tissues (pancreas and whole blood) obtained by bootstrapping genes (n = 1,000). Error bars are 95% confidence interval. **e**. Bootstrapped mean enrichment plots for all independent eQTLs in PBMC, pancreas, and pituitary for closely linked tissues obtained using bootstrapping (n = 1,000). Error bars indicate 95% confidence interval. Two-sided bootstrapped *P* values are plotted (**b**–**e**). **f**,**g**. Enrichment plots for RegulomeDB variants with ranking=1a in (**f**) pancreas and (**g**) pituitary multi-ome.

Next, we assessed whether the enhancer tiles predicted by SCARlink can be used to prioritize genetic variants causally associated with gene regulation and disease etiology. To this end, we first filtered a set of gene-linked tiles for each gene and cell type based on the significance of an approximate Shapley score (Methods; Fig. 3a). We observed that these predicted gene-linked tiles were sensitive to the sparsity of the dataset (Extended Data Fig. 5a). We then performed an enrichment analysis of the resulting set of gene-linked tiles with respect to statistically fine-mapped eQTLs (posterior inclusion probability (PIP) > 0.5) for the corresponding genes in the closest matched GTEx tissues^15^, and with respect to 17,769 statistically fine-mapped GWAS variants (PIP > 0.2) across 82 UK Biobank traits^16^ (average, *n* = 334,803; Supplementary Table 3) in PBMC, pancreas and pituitary gland (Methods). SCARlink gene-linked tiles in the three multi-ome datasets show 5.5× to 7.5× enrichment of fine-mapped GWAS variants with respect to a set of common variants matched by linkage disequilibrium (LD), minor allele frequency (MAF) and gene distance in the top 15,000 predicted gene-linked tiles (Supplementary Table 4) and outperformed a standard pairwise peak–gene linking implemented by ArchR. The enrichment increases with higher PIP thresholds (Fig. 3b). Moreover, the enrichment of the GWAS variants is individually the same or higher for 79% of the 82 traits in SCARlink gene-linked tiles (Extended Data Fig. 5b). Upon subsetting by distance annotations, SCARlink enrichment is equal or higher than that of ArchR for a large fraction of traits in promoter-proximal and distal regions (Extended Data Fig. 5b).

Next, we performed comparative disease heritability analysis of SCARlink-linked tiles/peaks and ArchR-linked peaks for the same set of genes using the stratified LD score regression (S-LDSC) method^17,18^; we assessed performance using the heritability enrichment and standardized effect size (τ*) metrics (Methods). In our marginal analysis, conditional on 97 baseline-LD (v2.2) annotations comprising of coding, conserved, epigenomic and LD-related annotations, SCARlink showed 1.2× higher meta-analyzed heritability enrichment compared to ArchR across 104 diseases and traits; results were concordant when conditioning on 53 baseline annotations^19^ and 17 LD- and MAF-related annotations^20^ (Methods; Extended Data Fig. 5c and Supplementary Tables 5 and 6). Conditional on the MAF- and LD-related annotations, SCARlink exhibited higher meta-analyzed standardized effect size (τ* = 0.67, *P* = 7 × 10^−27^) compared to ArchR (τ* = 0.58, *P* = 2 × 10^−30^); however, this disease signal was not significant conditional on the baseline and baseline-LD annotations (Extended Data Fig. 5c). Next, we performed a joint heritability analysis of SCARlink and ArchR annotations. Conditional on the MAF- and LD-related annotations, both SCARlink and ArchR annotations showed jointly significant τ*; however, SCARlink showed 1.9× higher joint disease information. Based on these results, we conclude that SCARlink predictions are more disease informative compared to ArchR annotations, based both on the enrichment of fine-mapped variants and disease heritability analyses.

For the fine-mapped eQTL traits from matched GTEx tissues, we observed 12× to 20× enrichment in PBMC for the first 20,000 gene-linked tiles (Fig. 3c, left) and 10× enrichment across predicted gene-linked tiles at FDR < 0.001 (Fig. 3c, right). We also observed 15× enrichment in pancreas multi-ome. Both PBMC and pancreas multi-ome gene-linked tiles have substantially higher enrichment than the enrichment using ArchR gene-linked peaks (Fig. 3c). To assess tissue-specific eQTL enrichment, we calculated the enrichment in PBMC and pituitary multi-ome of eQTLs from nonmatching tissues from the GTEx database. We observed lower enrichment of eQTLs from other GTEx tissues (Fig. 3d and Extended Data Fig. 5d), suggesting that SCARlink can identify variants in regulatory regions that are tissue-specific and cell-type-specific.

We then assessed the enrichment of SCARlink gene-linked tiles in conditionally independent eQTL signals from GTEx. SCARlink showed 10× to 21× enrichment of primary eQTLs (defined by the eQTL with the most significant association for the gene) in the pancreas for the top 4,000 predicted gene-linked tiles and substantially higher enrichment in PBMC compared to ArchR peaks (Fig. 3e and Extended Data Fig. 5e). We additionally performed the enrichment analysis of SCARlink gene-linked tiles with different categories of variants from RegulomeDB^21,22^. SCARlink showed higher enrichment for the top 20,000 gene-linked tiles in PBMC over ArchR peak–gene links for 5,461 RegulomeDB variants with a rank of 1a, corresponding to the most stringent cutoff based on motif accessibility at eQTL/caQTLs (Fig. 3f). SCARlink tiles also show higher enrichment for the top 4,000 gene-linked tiles in pancreas and top 12,000 tiles in pituitary (Extended Data Fig. 5f,g and Supplementary Table 7).

### Predicted enhancers prioritize disease-associated loci in a cell-type-specific manner

**Fig. 4:**
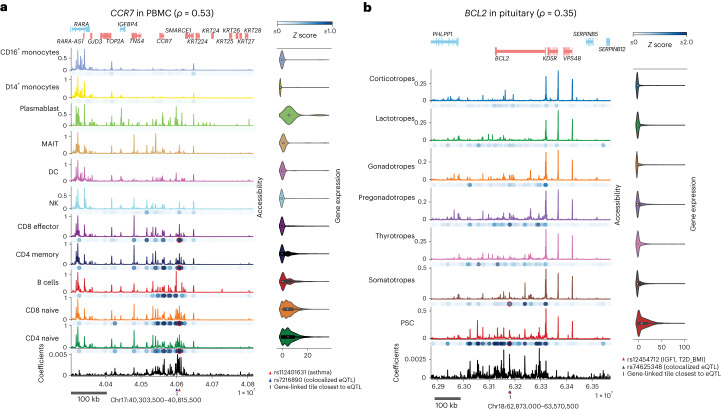
SCARlink-derived gene-linked tiles can reveal cell-type-specific disease-gene associations across tissues. **a**, SCARlink output of CCR7 in PBMC (*n* = 9,460 cells; Supplementary Table 1). The red triangle denotes a fine-mapped GWAS variant associated with asthma. The same position is highlighted in red under the cell types for which SCARlink predicted the variant-containing tile to be important. The blue triangle marks the colocalized eQTL (PP.H4 = 0.9022; coloc^23^) and the vertical line below highlights the tile closest to the eQTL substantially linked to CCR7. **b**, SCARlink output of BCL2 in the pituitary (*n* = 11,549 cells; Supplementary Table 1). The red triangle at the bottom denotes a variant associated with IGF-1 and T2D_BMI. The tile containing the variant is highlighted in red for cell types for which SCARlink predicted the tile to be important. The blue triangle marks the colocalized eQTL (PP.H4 = 0.9456; coloc^23^) and the vertical line below highlights the tile closest to the eQTL substantially linked to BCL2. *ρ* indicates the Spearman correlation between predicted and observed gene expression. The boxplots inside the violin plots for gene expression in **a**and **b** are centered on the median, bounded by the quartiles, with the whiskers depicting the remaining distribution. *Z*scores in **a** and **b** correspond to the cell-type-specific-standardized Shapley values.

We also examined variants causally linked to disease and gene expression phenotypes based on GWAS and eQTL studies and used SCARlink to link the variant-containing tile to the gene in specific cell types. One such variant is rs112401631 (chr17:40608272:T:A), a fine-mapped variant for asthma (PIP = 0.27) that colocalizes with an eQTL chr17:40600717:G (PP.H4 = 0.9022; coloc^23^) linked to *CCR7* gene in the lymphoblastoid cell line^23,24^. The *CCR7* gene is well-known for its role in the homing of T cell populations to lymphoid organs^25,26^, and CCR7^+^ memory CD4^+^ T cells have previously been associated with severity of asthma^27,28^. In PBMC data, the tile underlying rs112401631 was predicted to be significantly linked to the *CCR7* gene in various T cell subtypes (CD8 effector with FDR-adjusted *P* = 3.7 × 10^−08^, CD4 memory with FDR-adjusted *P* = 5.9 × 10^−21^, CD8 naive with FDR-adjusted *P* = 7.6 × 10^−18^and CD4 naive with FDR-adjusted *P* = 2.2 × 10^−17^; Fig. 4a). Although the eQTL-containing tile is not predicted to be linked to *CCR7* by SCARlink, the tile 783 bases from the eQTL is significantly linked in CD8 naive T cells (FDR-adjusted *P* = 2.69 × 10^−07^; Supplementary Table 8). This tile includes the variant rs1358175 that is in LD with the colocalized eQTL (*R*^2^ = 1; Supplementary Table 8). Furthermore, the 10 kb window around the GWAS causal variant contains enhancers exclusive to the T cell subsets (Supplementary Table 9). Based on these results, we hypothesize that SCARlink-predicted links can be used to ascertain putatively causal cell types underlying GWAS and eQTL colocalizations.

**Extended Data Fig. 6:**
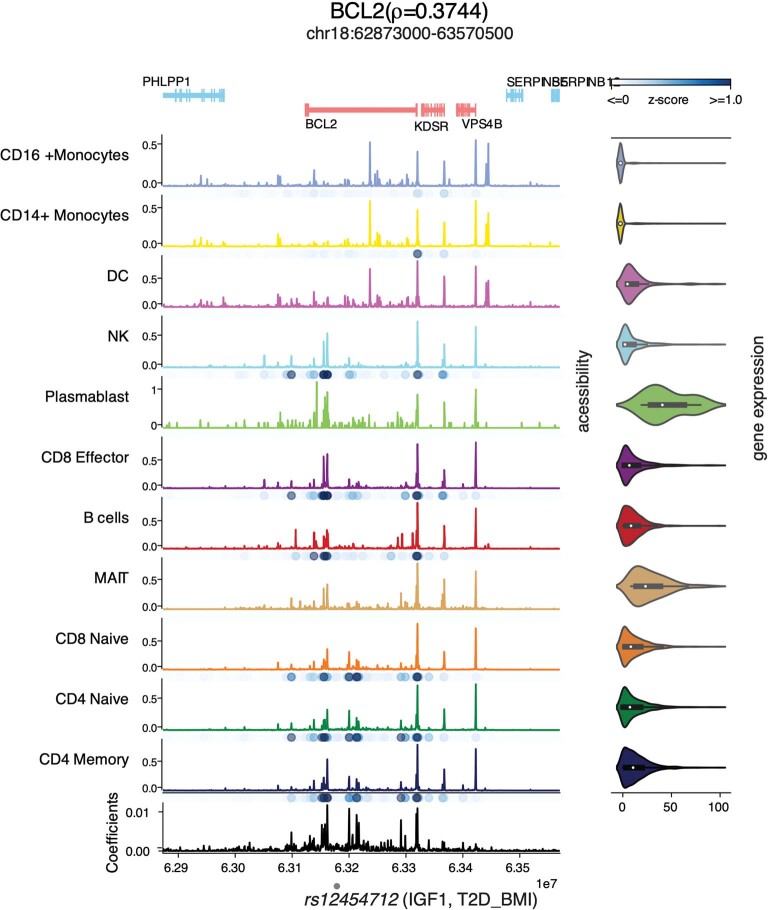
SCARlink output of BCL2 in 10x PBMC. The gray dot at the bottom denotes the variant associated with IGF1 and T2D_BMI. The variant-containing tile is not important across any of the cell types in PBMC (n = 9,460 cells, Supplementary Table 1). The boxplots inside the violin plots for gene expression are centered on the median, bounded by the quartiles, with the whiskers depicting the remaining distribution. Z scores correspond to the cell-type-specific standardized Shapley values.

A second example is the fine-mapped variant rs12454712 (chr18:63178651:T:C) for concentrations of circulating insulin-like growth factor 1 (IGF-1; PIP = 0.99) and type 2 diabetes (adjusted by BMI; PIP = 0.99) and lies in an intronic enhancer of *BCL2*(ref. ^29^). IGF-1 is known to prevent apoptosis through the activity of *BCL2*, which encodes an antiapoptotic transcription factor^30^. Furthermore, somatotropes, endocrine cells in anterior pituitary, secrete growth hormone that affects the production of IGF-1 and IGF-1 in turn negatively regulate growth hormone production^31^. Interestingly, we found this variant to be in a regulatory region of pituitary stem cells (PSCs; FDR-adjusted *P* = 1.3 × 10^−10^) and somatotropes (FDR-adjusted *P* = 6.3 × 10^−04^; Fig. 4b), possibly suggesting a role in pituitary stem cell differentiation. Additionally, both high and low IGF-1 levels have been associated with insulin resistance and a higher risk of type 2 diabetes, respectively^32^. While we found this variant within the regulatory region of cells from the pituitary gland, it is not accessible in the PBMC multi-ome (Extended Data Fig. 6), and SCARlink appropriately assigns the tile low significance in these cell types. Moreover, GWAS-eQTL colocalization analysis reveals that the eQTL chr18:63179197:G (PP.H4 = 0.9456; coloc^23^) lies in a tile substantially linked to *BCL2* in PSCs (Supplementary Table 8).

### SCARlink-predicted gene expression enables chromatin potential analysis

We next asked whether SCARlink-identified regulatory regions become accessible before transcription of the modeled genes in developmental settings and thus can be used to determine the developmental trajectory through chromatin potential^1,9^. This method can be viewed as computing the arrow direction in a cell embedding of the neighboring cells having the most similar observed gene expression to the current cell’s predicted expression. The arrows are then plotted on the same cell embedding to visualize the differentiation trajectory. Analogous to the original definition of chromatin potential-based correlation between DORCs and genes, we computed a smoothed SCARlink-predicted gene expression vector for each given ‘source’ cell, identified a set of ‘target’ cells whose smoothed observed gene expression vectors are most correlated with the predicted source cell expression vector, determined the corresponding chromatin potential vector from the source cell toward the average position of the target cells and visualized in an FDL or UMAP embedding (Methods). We applied SCARlink in this fashion to derive chromatin potential vector fields for mouse skin, BMMC, pituitary gland and developing human cortex. When computing chromatin potential, by default, we chose all genes among the top 2,000 highly variable genes for which SCARlink-predicted gene expression was positively correlated with observed gene expression. This filtered out less than 5% of genes for mouse skin (19 of 434 genes), BMMC (36 of 785 genes) and pituitary gland (2 of 612 genes), and 6% of genes from the developing human cortex (73 of 1,201 genes).

**Fig. 5:**
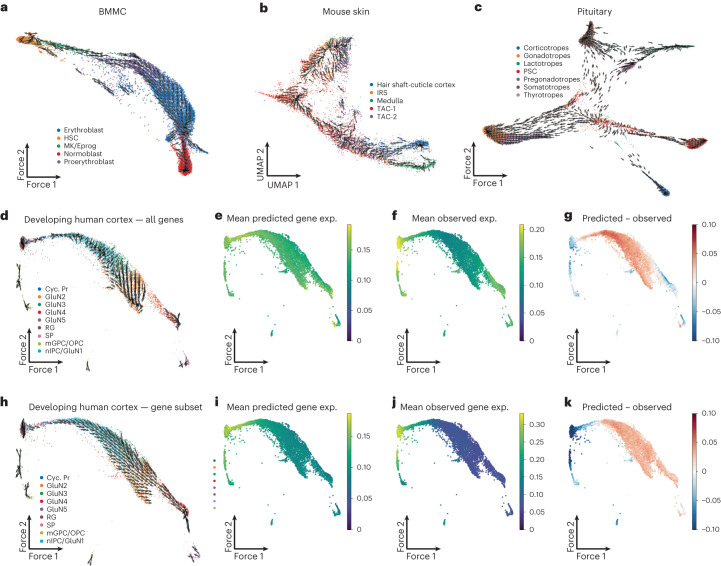
SCARlink provides a robust implementation of chromatin potential. **a**–**c**, SCARlink-computed chromatin potential applied to BMMC^8^ (7,155 cells and 785 genes; **a**), mouse skin^1^ (6,431 cells and 434 genes; **b**) and pituitary gland^12^ (11,549 cells and 1,221 genes; **c**) recapitulates known differentiation trajectory in each system. The arrows point toward the direction in which the observed gene expression is most similar to SCARlink-predicted gene expression. **d**, Chromatin potential does not capture the known differentiation trajectory of developing human cortex^9^ (4,642 cells and 1,221 genes) when using all genes based on the correlation of predicted and observed gene expression. **e**–**g**, For the genes used in **d**, plots show the mean SCARlink-predicted expression (**e**), the mean observed expression from scRNA-seq (**f**) and the difference between the mean predicted and observed expression (**g**). **h**, The known trajectory of the developing human cortex is better represented when only using a subset of the genes (470 genes). **i**–**k**. For the genes used in **h**, plots show the mean predicted expression (**i**), the mean observed expression (**j**) and the difference between the mean predicted and observed expression (**k**).

**Extended Data Fig. 7:**
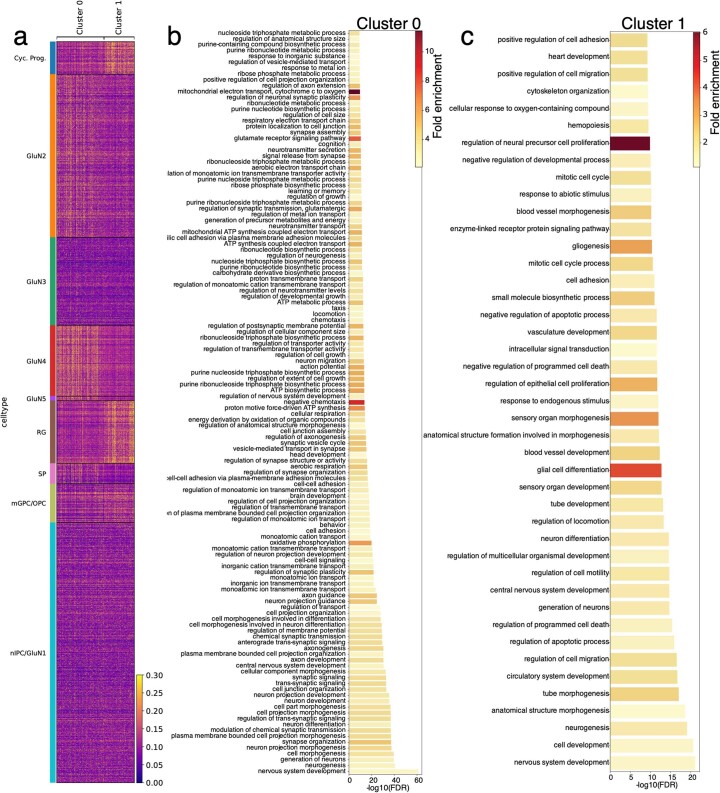
Enriched GO terms for the two clusters of genes in developing human cortex. **a**. Heatmap showing comparison of scaled gene expression from scRNA-seq in the two identified clusters across the cell types of developing human cortex (4,642 cells). **b**,**c**. Significant GO terms enriched in (**b**) cluster 0 (731 genes) and (**c**) cluster 1 (470 genes), colored by fold enrichment. Genes in cluster 1 capture the known differentiation trajectory.

**Extended Data Fig. 8:**
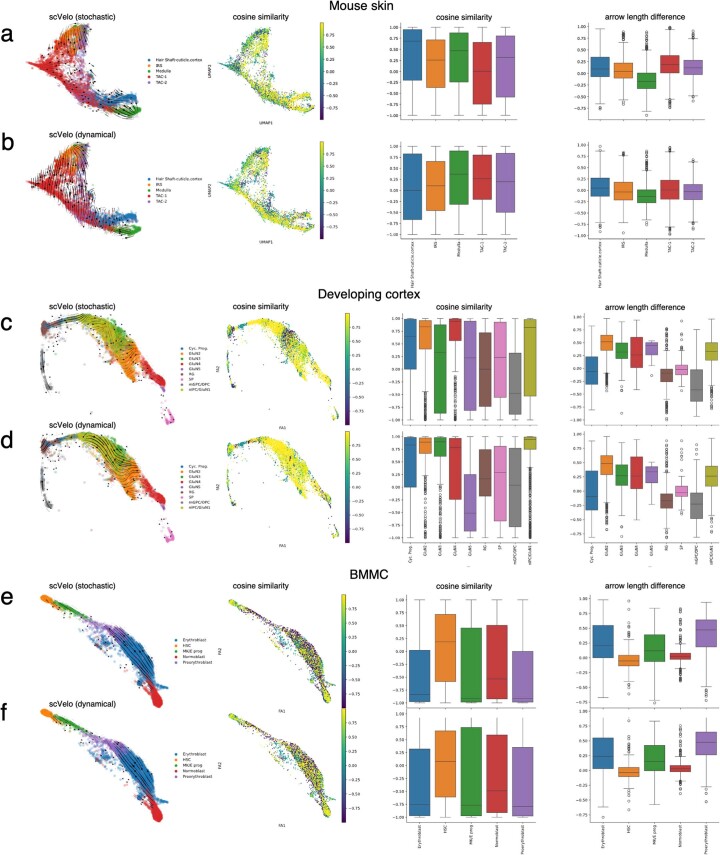
Comparison of chromatin potential to RNA velocity. RNA velocity was estimated using scVelo^56^ on mouse skin^1^ (**a**,**b**) (n = 33,314 cells, Supplementary Table 1), developing cortex^9^ (**c**,**d**) (n = 4,642 cells, Supplementary Table 1), and BMMC^8^ (**e**,**f**) (n = 7,155 cells, Supplementary Table 1). scVelo was run in two modes: ‘stochastic’ mode with the previously reported gene set (**a**,**c**,**e**) or with chromatin potential genes (**f**), and ‘dynamical’ mode with the previously reported gene set (**b**,**d**). The direction of velocity (first column) changes between the two modes (**a**–**d**) and the direction is opposite to known trajectory in BMMC (**e**,**f**). The scVelo arrows (first column) depict the direction of RNA velocity based on spliced and unspliced mRNA counts. Cosine similarity between scVelo arrows and chromatin potential arrows are plotted on UMAP (second column) or grouped by cell type (third column). The difference in the length of chromatin potential and RNA velocity arrows are grouped by cell type (fourth column). The boxplots are centered on the median, bounded by the quartiles, with the whiskers extending up to values within 1.5× inter-quartile range (IQR), and the remaining points as outliers.

We found that the SCARlink chromatin potential vector fields recapitulate known differentiation trajectories in mouse skin, BMMC and pituitary gland (Fig. 5a–c). However, in developing human cortex cells, chromatin potential failed to identify that the radial glia cell population is the root cell type^9^ (Fig. 5d). Upon comparing the difference between predicted and observed gene expression averaged over all genes, we found that this difference is the highest in the middle of the known developmental trajectory (nIPC/GluN1) and decreases afterward (Fig. 5d–g). Examining further, we identified two clusters of genes based on hierarchical clustering of single-cell expression patterns (Extended Data Fig. 7a and Supplementary Table 10), with one cluster enriched for gene ontology terms related to glial cell differentiation (Extended Data Fig. 7b,c). Performing SCARlink chromatin potential analysis on this subset of 470 genes recovered the correct developmental trajectory (Fig. 5h). For this subset of genes, we also found that the difference between average predicted and observed gene expression increases over the course of the trajectory, consistent with the opening of chromatin at these loci preceding target gene expression (Fig. 5i–k). While our analysis demonstrates the utility of chromatin potential as a strategy to identify a differentiation trajectory in multi-ome datasets, we also caution that prior selection of a subset of genes may be required to obtain results consistent with known biology. Furthermore, as reported previously^1^, we found that chromatin potential often identified developmentally correct trajectories in settings where RNA velocity performed inconsistently or failed (Extended Data Fig. 8).

## Discussion

We have shown that SCARlink provides an effective and robust method for identifying cell-type-specific enhancers of genes without prior computation of a peak set. SCARlink also efficiently resolves the cell-type specificity of tissue-relevant eQTLs and GWAS traits using Shapley value analysis and computes chromatin potential vector fields tracking development or differentiation.

We note that SCARlink is designed to be a simple gene-level model, namely a (regularized) generalized linear model with a log link function and constrained to have non-negative regression coefficients. This simplicity enables fast training and model selection for predicting gene expression as well as very efficient computation of approximate Shapley values to identify significant tiles in a cell-type-specific manner.

The imputed gene expression estimated by SCARlink also enables the computation of chromatin potential from multi-omic data. Additionally, by modeling additive positive effects, we obtain a highly interpretable model where significant tiles from Shapley analysis are validated by chromosome conformation capture data and enriched for fine-mapped eQTLs and GWAS variants. We also expect that SCARlink’s cell-type-specific enhancers and enhancer–gene links could be incorporated into functionally driven transcriptome-wide association study (TWAS) methods for predicting gene expression from genotypes^33–36^.

Despite the effectiveness of SCARlink’s generalized linear modeling, we can anticipate settings where more complex gene-level models might be suitable; for example, one could include interaction terms between tiles in the regression model or even employ nonlinear neural network architectures for the same single-cell gene expression prediction task. Our implementation of SCARlink in TensorFlow should facilitate the implementation of and comparison to these more complex models.

Finally, there has been extensive work on DNA sequence models for bulk epigenomic and scATAC–seq data^37,38^, including in the context of the prediction of bulk gene expression^39,40^. In future studies, we plan to integrate DNA sequence information into SCARlink, sharing the sequence model associated with each cell across gene models, with the goal of modeling the regulatory grammar in enhancers as well as their regulatory impact on target gene expression.

## Methods

### Ethics statement

This study did not generate any biological samples and used publicly available datasets.

### Data preprocessing

Single-cell multi-omic data were processed using Seurat (v4.3)^41^; scRNA-seq) and ArchR (v1.0.2)^6^; scATAC–seq). We performed quality control separately for scRNA-seq and scATAC–seq. We filtered out cells with mitochondrial reads >20% for scRNA-seq with unannotated cell types (10× PBMC and pancreas). For scATAC–seq, we filtered for cells with at least 1,000 fragments and performed doublet detection on unannotated datasets. For doublet detection, we first estimated doublet scores using the function addDoubletScores() from ArchR and then filtered doublets with filterDoublets() from ArchR. ArchR also splits the genome into tiles of a specific size (500 bp by default) and computes the Tn5 insertion counts for each tile. The insertion counts are set to zero for blacklisted regions by ArchR. We performed counts per 10,000 normalizations of the scRNA-seq data. Then we ordered the cells in the same manner for both scRNA-seq and scATAC–seq. We selected the top 5,000 highly variable genes, using Seurat, and used this gene set as input to SCARlink.

### Cell-type annotation

Cell-type annotation was provided by the original studies for BMMC, developing human cortex and mouse skin. We clustered and annotated the clusters of the pituitary gland dataset based on previously reported marker genes^42^ (Supplementary Tables 11 and 12). We clustered the pancreas data and separately annotated the cells with the help of a pancreas reference atlas^43^ using the following label transfer functions of Seurat^41,44^: FindTransferAnchors() and TransferData() (Supplementary Table 13). Then we renamed the cluster with the cell-type annotation having the maximum overlap with the cluster. If multiple cell-type annotations overlapped with a single cluster, we denoted that cluster as having ‘mixed’ cell type. In the case of the PBMC dataset, we used the PBMC marker genes from Azimuth^41^ (Supplementary Table 14) for annotating the clusters.

### Gene regression model

SCARlink uses regularized Poisson regression to predict single-cell gene expression from single-cell chromatin accessibility. This method can be applied to both single-cell and single-nucleus multi-ome data.

We used ArchR to split the genome into 500 bp tiles and computed tile-level scATAC–seq feature accessibility. We selected tiles that span 250 kb upstream/downstream of and across the gene body. The accessibility within the tiles was normalized by the ReadsInTSS parameter, which is also the default normalization in ArchR, to control for sequencing depth and sample quality^6^. Gene expression values were normalized by counts per million. For each gene, the chromatin accessibility input to SCARlink was ReadsInTSS-normalized, then min–max scaled on a per-tile basis. The min–max scaling is performed on the training cells and then the same learned rule is applied to scale the tile counts of the test set. We ran the model separately on the 5,000 most variable genes determined using Seurat. Additionally, we filtered out genes for which the expression was too sparse with a threshold of 0.9, or 90% zeros.

**Extended Data Fig. 9:**
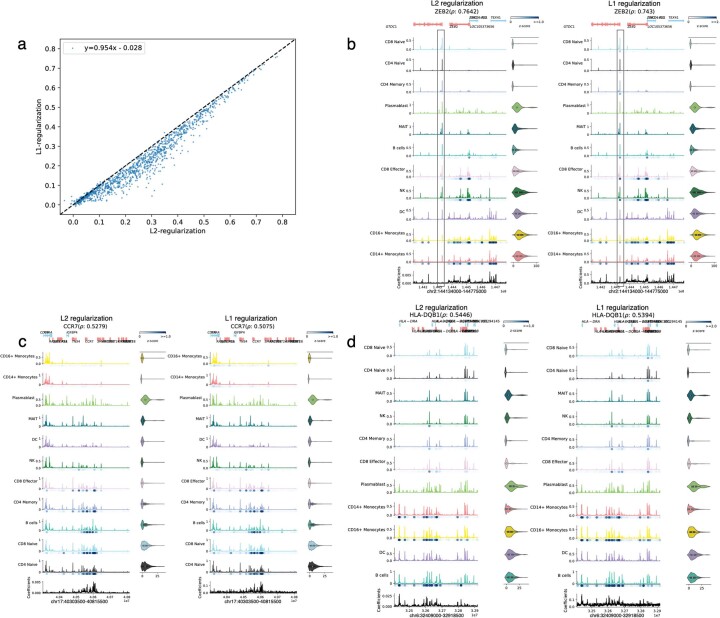
L2-regularization makes better predictions than L1-regularization. **a**. Comparison of Spearman correlations of predicted and observed gene expression for models trained using Poisson regression with L2 (x-axis) and L1 (y-axis) regularization on PBMC multi-ome (n = 9,460 cells, 1,241 genes, Supplementary Table 1). Each dot corresponds to a single gene. **b**–**d**. Comparison of learned regression coefficients when using L2 (left) and L1 (right) regularization for (**b**) ZEB2, (**c**) CCR7, and (**d**) HLA-DQB1. L1 regularization incorrectly assigns high regression coefficient to tile accessible across all cell types (highlighted box in **b**). L1 regularization fails to shrink most regression coefficients in (**c**,**d**). The boxplots inside the violin plots for gene expression in **b**–**d** are centered on the median, bounded by the quartiles, and with whiskers depicting the remaining distribution. Linear least-squares regression was performed to determine slope and intercept. Z scores correspond to the cell-type-specific standardized Shapley values.

We used regularized Poisson regression to predict gene expression from the tile matrix. L1 regularization results in poorer prediction of gene expression (Extended Data Fig. 9a) and the learned regression coefficients can have varying degrees of sparsity that lack interpretability (Extended Data Fig. 9b–d). Additionally, since training an elastic net would require training more models with different pairs of regularization parameters for L1 and L2 losses, and given the suboptimal results of L1 regularization, we did not consider using an elastic net approach. Thus, L2 regularization is used because it is preferable for prediction problems, and we are not filtering any features during model training.

For every gene, we optimized the following loss function:

$$\frac{1}{N}\mathop{\sum }\nolimits_{i=1}^{N}\left(e^{{X}_{i}{\mathbf{w}}+\epsilon}-{{Y}}_{i} \left({X}_{i}{\mathbf{w}}+\epsilon \right)\right)+\alpha {\left\Vert{\mathbf{w}}\right\Vert}_{2}^{2}$$

Here *n* corresponds to the number of cells, *X* corresponds to the min–max scaled accessibility matrix, **y** corresponds to the gene expression vector, **w** is the learned regression coefficient vector and *α* is the regularization parameter. We left out one-fourth of the data for testing. The regularization parameter was selected using fourfold cross-validation on the remaining three-quarters of the cells. The Spearman correlation was computed on the held-out test cells. We used TensorFlow in Python to develop the model and the Adam optimizer for training. We constrained the regression coefficients to be non-negative, thereby learning only positive regulators for genes.

### Significance test for model predictions on individual genes

To compare the overall performance of SCARlink predictions on test cells with other methods based on the Spearman correlation with ground truth, we used a Wilcoxon signed-rank test over genes.

We also estimated whether the Spearman correlations of SCARlink predictions are substantially different from the correlations using other methods for individual genes. The correlations from the two methods are not independent because they are calculated on the same observed gene expression values. We calculated the following test statistic for each gene and performed a *t*test to estimate significance (one-sided)^45^:

$$t=\left(\,{\rho }_{12}-{\rho }_{13}\right)\sqrt{\frac{(n-1)(1+{\rho }_{23})}{2\left(\frac{n-1}{n-3}\right){\rm{|}}S{\rm{|}}+\frac{{\left(\,{\rho }_{12}+{\rho }_{13}\right)}^{2}}{4}{\left(1-{\rho }_{23}\right)}^{3}}} \sim T(n-3)$$

where$,\left|S\right|=1-\left({\rho }_{12}^{2}+{\rho }_{13}^{2}+{\rho }_{23}^{2}\right)+2{\rho }_{12}{\rho }_{13}{\rho }_{23}$, *ρ*_12_ is the Spearman correlation between SCARlink prediction and observed gene expression, *ρ*_13_ is the Spearman correlation between ArchR gene score/DORC score prediction and observed gene expression, *ρ*_23_ is the Spearman correlation between SCARlink prediction and ArchR gene score/DORC score prediction and *n* is the number of cells in held-out test set.

We performed FDR correction of the *P* values using the Benjamini–Hochberg method^46^. The scatter plots in Fig. 1b–e and Extended Data Fig. 3a–c are colored using these FDR-corrected *P* values.

### Shapley scores and tile significance

After training the model, we used the SHAP Python package (v0.41.0)^47^ to compute Shapley values for a linear model, which closely approximate the Shapley values of our Poisson regression model.

$${\rm{sha}}{{\rm{p}}}_{{{t}}}={{{W}}}_{{{t}}}\left({{{X}}}_{* ,{{t}}}-{\rm{mean}}\left({{{X}}}_{* ,{{t}}}\right)\right)$$

Here shap_*t*_ corresponds to the Shapley value of a particular tile *t*.

We computed these approximate Shapley values in a cell-type-specific manner. For each cell type, we iteratively sampled 50 training cells from the cell type to form a pseudobulk sample and computed Shapley values for each tile of the pseudobulk profile. We iterated 500 times and then averaged the Shapley values for each tile over iterations. This gave an average Shapley score for each tile and cell type. Finally, we standardized the scores using*z*-score transformation. We scaled features this way separately for each gene model to identify gene-linked tiles. Note that we estimate Shapley values only for cell types having at least 100 cells.

### PCHi-C analysis

We used publicly available PCHi-C data for hematopoietic cells^14^. We transformed the coordinates from hg19 to hg38 with LiftOver^48^. PCHi-C loops at each promoter bait were identified by fitting a negative binomial generalized additive model^49^ to the observed counts as a function of GC content, mappability and length of the restriction fragments alongside a smooth distance function parameterized using a reduced-rank thin plate spline basis using the GAMLSS R package. If replicates were present, a replicate covariate was added to the model to control for library size. After this base model was fit, interactions were flagged by using the fitted distributions to compute a *P* value. This overall strategy is akin to the GLM-based strategy of HiC-DC+ to identify significant interactions^50^. After *P* values were computed for each restriction fragment in the vicinity of a promoter bait, *P* values across replicates were pooled using Fisher’s method and corrected using Benjamini–Hochberg for each promoter bait. To further improve our ability to detect interactions, we employed locally adaptive weighting and screening to smooth the *P* values and simultaneously control for the false discovery rate^51^.

For the Shapley value comparison, we used the AverageExpression function from Seurat^41^ to calculate average scaled gene expression and selected highly expressed genes per cell type. For every cell type, we restricted to genes with an average scaled gene expression of more than 0. Then, we chose the top 50 genes if there were more than 50 highly expressed genes per cell type. Next, we extracted all tiles that contain significant PCHi-C interactions for CD4 naive T, CD8 naive T, CD8 memory T and B cells for these genes. If there were multiple tiles spanning one PCHi-C interaction, we selected the maximum Shapley value across the tiles. The background Shapley values are from tiles that do not contain any significant PCHi-C interactions for the same genes. Next, we extracted tiles with accessibility similar to the accessibility within peaks. We used an accessibility threshold greater than 10% of least accessible peaks. This ensured that both PCHi-C interaction containing tiles and background tiles are accessible. We further subsampled tiles from both sets to ensure similar accessibility distribution. We performed the Mann–Whitney *U* test per cell type to assess the significance of the difference in Shapley scores between tiles with/without PCHi-C interactions.

### ArchR peak2gene

We used ArchR^6^ to first perform peak calling using MACS2 (v2.2)^52^ grouped by the cell-type annotations. We then used the ArchR pipeline to link peaks to genes, which performs a pairwise correlation of accessibility and gene expression on aggregated meta-cells. We used the same genomic window as SCARlink to predict the peak–gene links.

### Tile significance for variant analysis

We found that the scaled Shapley scores were not comparable across gene models. Therefore, we used an additional metric to order the gene-linked tiles when computing enrichment–recall curves; specifically, we estimated the significance of difference in the prediction of gene expression with and without a specific tile on held-out test cells using a paired Wilcoxon (signed-rank) test. We performed this significance test in a cell-type-specific manner across all genes in each multi-ome dataset. The resulting *P* values were then FDR-corrected using the Benjamini–Hochberg method^46^.

### GWAS enrichment analysis

**Extended Data Fig. 10:**
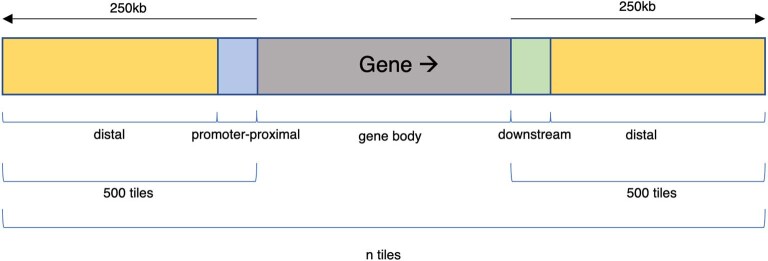
Genomic annotation for the tiles of each gene model. Distance-based annotation of the tiles to select common variants for enrichment calculation. Here the example gene is depicted to transcribe from right to left. For genes on the opposite strand, the promoter and downstream annotated regions are flipped.

We used fine-mapped GWAS variants from UK Biobank (Data availability) and first filtered out variants that lie within exons or are splicing eQTLs. The exons were extracted from hg38 RefSeq annotations from UCSC genome browser^53^. UK Biobank originally had 94 traits. We retained the top 90% of the traits based on the number of fine-mapped variants lying within 250 kb of all the genes SCARlink was trained on. This resulted in 82 traits. We considered a variant to be a causal variant if it is associated with at least one trait with PIP > 0.2. This resulted in 17,769 fine-mapped causal variants that are present in tiles spanning 250 kb upstream/downstream of all the genes from PBMC, pancreas and pituitary. Next, for each trait, we ensured that the common variants and causal variants were matched based on the following criteria (Extended Data Fig. 10): (1) the same minor allele frequency (MAF) category. We defined MAF groups as <0.01, 0.01–0.1 and >0.1; (2) the same LD blocks as defined in https://github.com/jmacdon/LDblocks_GRCh38/blob/master/data/pyrho_EUR_LD_blocks.bed; (3) the same distance-based genome annotations. Because we ran SCARlink with 500 bp tiles that span 250 kb upstream and downstream of the gene body, the first 500 and last 500 tiles correspond to the flanking upstream/downstream regions. To make the distance annotation computationally less expensive, we labeled the middle tiles, spanning from the 500th tile to the *n*-500th tile, as ‘gene body’, the 20 tiles upstream (left or right of the gene body depending on the strand) of the gene as ‘promoter-proximal’ corresponding to 10,000 bases and the 20 tiles downstream of the gene as ‘downstream’ corresponding to the downstream 10,000 bases. The remaining tiles are annotated as ‘distal’ regions. Here *n*stands for the total number of tiles for a given gene model. Note that for all genes, the number of tiles annotated as ‘promoter/upstream’, ‘downstream’ and ‘distal’ will be the same.

For each trait *i*, and matched group *g* of MAF, LD block and genome annotation, we calculated precision as the ratio of the number of causal variants in predicted gene-linked tiles/peaks to the number of common variants in predicted gene-linked tiles/peaks. Then, we calculated enrichment as described previously^2^, by dividing precision by the probability of encountering a causal variant of the given trait across all the tiles. We finally computed the average enrichment across all the traits as follows:

$$\begin{array}{l}{\rm{{Precisio}{{n}_{{trait}}}}}_{i,g}\\=\displaystyle\frac{{\rm{number}\, {of}}\,{\rm{{causal}\,{variants}\,{of}\,{trai}{t}}}_{i}\,{{\rm{in}\,{group}}}\,g\,{{\rm{of}\,{gene}\,{linked}\,{tiles}/{peaks}}}}{{\rm{number}\, {of}}\,{{\rm{common}\,{variants}\,{in}\,{group}}}\,g\,{{\rm{of}\,{gene}\,{linked}\,{tiles}/{peaks}}}}\end{array}$$

$$\begin{array}{l}{{\rm{Probability}}}\left({\rm{{causal}\,{variant}\,{of}\,{trai}{t}}}_{i,g}\right)=\\\displaystyle\frac{\begin{array}{l}{\rm{number}\,{of}}\,{\rm{{causal}\,{variants}\,{of}\,{trai}{t}}}_{i}\,{\rm{in}}\,{\rm{group}}\\g\,{\rm{across}}\,{\rm{all}}\,{\rm{tiles}}\,{\rm{of}}\,{\rm{SCARlink}}\,{\rm{genes}}\end{array}}{\begin{array}{l}{\rm{number}\,{of}}\,\rm{common}\,\rm{variants}\,\rm{across}\,\rm{all}\\\rm{tiles}\,\rm{from}\,\rm{group}\,g\,{{\rm{of}\,{SCARlink}\,{genes}}}\end{array}}\end{array}$$

$${\rm{Enrichmen}}{{\rm{t}}_{{\rm{trait}}}}_{i,g}=\frac{{\rm{Precisio}}{{\rm{n}}_{{\rm{trait}}}}_{i,g}}{{\rm{probability}}\left({\rm{causal}}\,{\rm{variant}}\,{\rm{of}}\,{\rm{trai}}{\rm{t}}_{i,g}\right)}$$

$${\rm{Enrichmen}}{\rm{t}}_{{\rm{trai}}{\rm{t}}_{i}}={\rm{average}}\left({\rm{Enrichmen}}{{\rm{t}}_{{\rm{trait}}}}_{i,g}\right)$$

$${\rm{Enrichment}}={\rm{average}}\left({\rm{Enrichmen}}{{\rm{t}}_{{\rm{trait}}}}_{i}\right)$$

In the case of SCARlink gene-linked tiles, we restricted to genes having SCARlink-predicted gene expression correlation of >0.1 and to gene-linked tiles with FDR < 0.001. For ArchR gene-linked peaks, we restricted to peaks having a correlation of >0.1 and FDR < 0.001.

### S-LDSC

Using a correlation cutoff value of 0.1 and FDR < 0.001, we obtained 1,730 genes in common to both SCARlink and ArchR peak2gene predictions from PBMC, pituitary and pancreas. To ensure comparable polygenicity, we used gene-linked tiles/peaks from the same 1,730 genes for S-LDSC. S-LDSC determines the contribution of a genomic annotation to disease and complex trait heritability^17,19^. For our analysis, the genomic annotations correspond to the SCARlink or ArchR predictions of gene-linked tiles/peaks along with baseline annotations related to LD, MAF, coding and epigenomic regions (Supplementary Table 5). We performed a marginal or joint analysis of the predicted gene-linked tiles/peaks conditional on the following three different baseline annotations: LD + MAF^18^ (17 annotations), baseline (53 annotations) and baseline-LD (97 annotations).

Briefly, S-LDSC considers the per-single-nucleotide polymorphism (SNP) heritability or variance of effect size (of standardized genotype on trait) of each SNP to be equal to the linear contribution of each annotation, $\mathrm{var}(\,{\beta }_{j})={\sum }_{j}{a}_{{cj}}\tau (c)$, where *a*_*cj*_ is the value of annotation *c* for SNP *j*, and *τ*(*c*) is the contribution of annotation *c* to per-SNP heritability conditioned on other annotations. S-LDSC estimates *τ*(*c*) for each annotation using the following equation:

$$E(\,{\chi }_{j}^{2})=n\sum _{{\rm{c}}}l\left(\;j,c\right)\tau \left(c\right)+1$$

where $l\left(\;j,c\right)={\sum }_{k}{a}_{{ck}}{r}_{{jk}}^{2}$ is the stratified LD score of SNP *j* with respect to annotation *c* and *r*_*jk*_ is the genotypic correlation between SNPs *j* and *k* computed using data from 1000 Genomes Project; *n* is the GWAS sample size. We assess the informativeness of a given annotation *c* using two metrics. The first metric is enrichment (*E*), defined as follows (for binary and probabilistic annotations only):

$$E={h}_{g}^{2}(c)/{h}_{g}^{2}\times M/\sum _{j}{a}_{{cj}}$$

where ${h}_{g}^{2}(c)$ is the heritability explained by the SNPs in annotation *c*, weighted by the annotation values. The second metric is standardized effect size (τ*) defined as follows:

$${\tau }^{* }(c)=\tau (c){\rm{s{d}}}_{c}/\left({h}_{g}^{2}/M\,\right)$$

where, sd_*c*_ is the standard error of annotation *c*, ${h}_{g}^{2}$ is the total SNP heritability and *M* is the total number of SNPs on which this heritability is computed (equal to 5,961,159 in our analyses). τ^*^ (*c*) represents the proportionate change in per-SNP heritability associated with a 1 s.d. increase in the value of the annotation.

### eQTL enrichment analysis

We used fine-mapped eQTLs from GTEx for whole blood, pancreas and pituitary for computing enrichment in gene-linked tiles/peaks. We defined causal variants as having PIP > 0.5. We filtered common variants for each gene based on matched MAF, LD and genomic annotations as described above. Then, separately for each gene, tissue and matched group *g*, we computed precision, enrichment and recall. We further computed the average enrichment and recall over genes per multi-ome dataset.

$$\begin{array}{l}{\rm{Precisio}}{{\rm{n}}_{{\rm{gene}}}}_{i,g}\\=\displaystyle\frac{\begin{array}{l}{\rm{number}}\, {\rm{of}}\,{\rm{{causal}}\,{\rm{variants}}\,{\rm{of}}\,{\rm{gene}}}_{\rm{i}}\,{\rm{in}}\\{{\rm{group}}}\,{\rm{g}}\,{{\rm{of}}\,{\rm{gene}}\,{\rm{linked}}\,{\rm{tiles}}/{\rm{peaks}}}\end{array}}{\begin{array}{l}{\rm{number}}\, {\rm{of}}\,{\rm{common}}\,{\rm{variants}}\,{\rm{in}}\,{\rm{tiles}}\,{\rm{from}}\,{\rm{group}}\,{\rm{g}}\,{\rm{around}}\\{\rm{gene}}_{\rm{i}}\,{\rm{in}}\,{\rm{gene}}\,{\rm{linked}}\,{\rm{tiles}}/{\rm{peaks}}\end{array}}\end{array}$$

$$\begin{array}{l}{\rm{Probability}}\left({{\rm{causal}}\,{\rm{variant}}\,{\rm{of}}\,{\rm{gene}}}_{{\rm{i}},{\rm{g}}}\right)\\=\displaystyle\frac{{\rm{number}}\, {\rm{of}}\,{{\rm{causal}}\,{\rm{variants}}\,{\rm{of}}\,{\rm{gene}}}_{\rm{i}}\,{\rm{in}}\,{\rm{group}}\,{\rm{g}}\,{{\rm{in}}\,{\rm{tiles}}\,{\rm{around}}\,{\rm{gene}}}_{\rm{i}}}{{{\rm{number}}\, {\rm{of}}}\,{{{\rm{common}}\,{\rm{variants}}\,{\rm{in}}\,{\rm{tiles}}\,{\rm{from}}\,{\rm{group}}}}\,{\rm{g}}\,{{{\rm{around}}\,{\rm{gene}}}}_{\rm{i}}}\end{array}$$

$${{\rm{Enrichmen}}{{\rm{t}}_{{\rm{gene}}}}}_{{\rm{i},{\rm{g}}}}=\frac{{\rm{Precisio}}{\rm{n}}_{{\rm{gene}}_{{\rm{i}},{\rm{g}}}}}{{\rm{probability}}\left({{\rm{causal}}\,{\rm{variant}}\,{\rm{of}}\,{\rm{gene}}}_{{\rm{i}},{\rm{g}}}\right)}$$

$${\rm{Enrichmen}}{\rm{t}}_{{\rm{gen}}{\rm{e}}_{\rm{i}}}={\rm{average}}\left({\rm{Enrichmen}}{{\rm{t}}_{{\rm{gene}}}}_{{\rm{i}},{\rm{g}}}\right)$$

$${{\rm{Enrichmen}}{\rm{t}}_{{\rm{gene}}}}={\rm{average}}\left({\rm{Enrichmen}}{{\rm{t}}_{{\rm{gene}}}}_{\rm{i}}\right)$$

Additionally, we performed a similar eQTL enrichment analysis on GTEx-independent eQTLs for whole blood, pancreas and pituitary. The primary independent eQTL is the most substantially associated variant^54^ and has a rank of 1. An eQTL with any other rank is an independent eQTL less important than the eQTLs with better ranks. There are at most 13 independent eQTLs per gene, and the whole blood sample has more nonprimary independent eQTLs than other tissues. We fixed a correlation cutoff value of 0.1 for both SCARlink genes and ArchR peak2gene links and FDR < 0.001.

### RegulomeDB enrichment analysis

The variants in RegulomeDB^21,22^ are assigned ranks based on their associated regulatory features. Each variant is also assigned a probability score based on a random forest model, where probability scores are correlated with the ranks. We chose the most stringent set of variants with a rank of 1a, corresponding to variants associated with eQTL/caQTL and TF binding with matched motif, footprint and accessible chromatin. We further restricted to variants with a probability score of >0.9. We considered these variants to be the putative regulatory variants.

We computed enrichment for each matched annotation and group *g* as follows:

$$\begin{array}{l}{{\rm{Precision}}}_{\rm{g}}\\=\displaystyle\frac{{\rm{number}}\, {\rm{of}}\,{{\rm{regulatory}}\,{\rm{variants}}\,{\rm{in}}\,{\rm{gene}}\,{\rm{linked}}\,{\rm{tiles}}/{\rm{peaks}}\,{\rm{for}}\,{\rm{group}}}\,{\rm{g}}}{{\rm{number}}\,{\rm{of}}\,{{\rm{common}}\,{\rm{variants}}\,{\rm{in}}\,{\rm{gene}}\,{\rm{linked}}\,{\rm{tiles}}/{\rm{peaks}}\,{\rm{for}}\,{\rm{group}}}\,{\rm{g}}}\end{array}$$

$$\begin{array}{l}{\rm{Probability}}\left({\rm{regulatory}}\,{\rm{variant}}\,{\rm{in}}\,{\rm{group}}\,{\rm{g}}\right)\\=\displaystyle\frac{{\rm{number}}\,{\rm{of}}\,{\rm{regulatory}}\,{\rm{variants}}\,{\rm{in}}\,{\rm{group}}\,{\rm{g}}}{{\rm{number}}\,{\rm{of}}\,{\rm{common}}\,{\rm{variants}}\,{\rm{in}}\,{\rm{group}}\,{\rm{g}}}\end{array}$$

$${\rm{Enrichment}}_{\rm{g}}=\frac{{\rm{Precision}}_{\rm{g}}}{{\rm{probability}}({\rm{regulatory}}\,{\rm{variant}}\,{\rm{in}}\,{\rm{group}}\,{\rm{g}})}$$

$${\rm{Enrichment}}={\rm{average}}({\rm{Enrichment}}_{\rm{g}})$$

### Downsampling analysis

We performed downsampling on the PBMC multi-ome. We downsampled accessibility counts in the tile matrices and the gene expression vectors on a per-cell basis using downsampleMatrix() from the R package, scuttle (v1.8.4)^55^. The downsampling was performed to generate sparse matrices with 33% and 66% of the original counts. For each of the 33% and 66% downsampled datasets, we ran SCARlink in the following manner: (1) downsampled scATAC–seq and original scRNA-seq, (2) original scATAC–seq and downsampled scRNA-seq and (3) downsampled scATAC–seq and downsampled scRNA-seq.

The resulting gene expression predictions were compared to the original input gene expression using the Spearman correlation. For each of the six SCARlink outputs, we predicted gene-linked tiles using the same cutoff values as the original PBMC output as follows: Spearman correlation > 0.1, standardized Shapley *z*score > 0.5 and FDR < 0.001.

Additionally, we performed downsampling of a number of cells and compared the predicted gene expression to the predictions by the original model. The number of cells was downsampled to 25%, 50% or 75% of the total number of cells in each dataset.

### Chromatin potential using SCARlink

We ran chromatin potential on smoothed SCARlink-predicted and observed gene expression values. Smoothing was performed over a *k*-nearest neighbor (kNN) graph (*k* = 50) built using a lower dimensional representation of the scATAC–seq data based on latent semantic indexing from ArchR. We retained the genes for which the predicted and observed gene expression are positively correlated. We then scaled the smoothed predicted and observed gene expression using min–max scaling. Following this, as in the published chromatin potential approach^1^, for each cell *i* in the predicted space, we identified the nearest neighbors (*k* = 10) in the observed space.

$${{{{Y}}}_{{\rm{obs}}}}_{i}={\rm{kNN}}\left({{Y}_{{\rm{pred}}}}_{i}\right)$$

Here ${{{{Y}}}_{{\rm{obs}}}}_{i}$ is the scaled and smoothed observed expression matrix of the ten cells with the highest correlation with the scaled and smoothed predicted expression vector of cell *i*, ${{Y}_{{\rm{pred}}}}_{i}$. We then plotted chromatin potential arrows on the UMAP or force-directed layout (ForceAtlas2 v0.3.5) from each cell *i*, to the average position of the cells corresponding to ${{{{Y}}}_{{\rm{obs}}}}_{i}$. These arrows are further smoothed over a grid layout on the FDL/UMAP embedding.

We used FDL visualizations for all datasets except mouse skin, where we used the previously published UMAP^1^. Additionally, for the mouse skin data, we ran the analysis on a subset of cell types to compare with the reported results^1^.

By default, we use the genes that are among the top 2,000 highly variable genes clearing the sparsity threshold. We do not filter out any genes except the ones with a negative correlation between predicted and observed expression. We found that by using the top 2,000 highly variable genes, we could not always obtain the known differentiation trajectory, as in the case of the developing human cortex. In this dataset, we performed hierarchical clustering of genes based on the cosine distance of observed gene expression vectors across all cell types, identified two clusters and repeated chromatin potential analysis with genes in one of the clusters.

### Comparison of chromatin potential and RNA velocity

We estimated RNA velocity using scVelo^56^. We downloaded preprocessed^57^ scVelo (v0.2.5) objects with spliced and unspliced genes for mouse skin and developing cortex and generated the spliced and unspliced counts for BMMC using velocyto^58^. We followed the standard scVelo workflow and estimated RNA velocity in ‘stochastic’ mode and ‘dynamical’ mode and visualized the output on the same UMAP or FDL used in Fig. 5a,b,d (Extended Data Fig. 8). Cosine similarity was used to compare the direction of arrows obtained from chromatin potential and RNA velocity. The difference in length of arrows obtained from chromatin potential and RNA velocity was computed and grouped by cell type to compare the magnitude of RNA velocity and chromatin potential for all stages of the differentiation trajectory.

### Statistics and reproducibility

No statistical method was used to predetermine the sample size. We ran our analysis on a subset of cells from samples showing the least batch effect as described in the Data availability. For each dataset, we selected the top 5,000 highly variable genes and then applied sparsity thresholding on gene expression before running SCARlink (Methods). In case of the S-LDSC analysis, we selected genes that had predicted gene-linked tiles/peaks for both SCARlink and ArchR peak2gene predictions. The experiments were not randomized, as all the datasets are publicly available from observational studies. The investigators were not blinded to allocation during experiments and outcome assessment, as the data were not from controlled randomized studies.

### Reporting summary

Further information on research design is available in the Nature Portfolio Reporting Summary linked to this article.

## Online content

Any methods, additional references, Nature Portfolio reporting summaries, source data, extended data, supplementary information, acknowledgements, peer review information; details of author contributions and competing interests; and statements of data and code availability are available at 10.1038/s41588-024-01689-8.

### Supplementary information

Reporting Summary

Supplementary Tables Supplementary Table 1: Comparison of SCARlink gene expression prediction to existing methods. Supplementary Table 2: Comparison of scaled Shapley scores of tiles with/without PCHi-C loops. Supplementary Table 3: UKBB traits used to calculate enrichment in PBMC, pancreas and pituitary multi-ome. Supplementary Table 4: UKBB causal variants in SCARlink gene-linked tiles. Supplementary Table 5: List of annotations and mean annotation values for each annotation in the three baselines used. Supplementary Table 6: S-LDSC results for marginal and joint models. Supplementary Table 7: RegulomeDB variants in SCARlink gene-linked tiles. Supplementary Table 8: Colocalization results for *CCR7* and *BCL2.* Supplementary Table 9: Gene-linked tiles predicted by SCARlink and lying within a 1 kb window around the GWAS variant, rs112401631, associated with asthma. Supplementary Table 10: Cluster labels for genes from developing human cortex multi-ome. Supplementary Table 11: Marker genes for pituitary cell types. Supplementary Table 12: Cell-type annotation for pituitary. Supplementary Table 13: Cell-type annotation for pancreas. Supplementary Table 14: Cell-type annotation for PBMC.

## Data Availability

We downloaded the PBMC multi-ome from 10X Genomics. BMMC data were part of the NeurIPS 2021 open problem, and the dataset was downloaded from GEO (GSE194122). We used BMMC samples labeled as site1_donor1, site1_donor2, site1_donor3, site2_donor1, site2_donor4, site2_donor5, site3_donor10, site3_donor6, site3_donor7 and site4_donor9 and the cell types HSC, MK/E progenitor, proerythroblast, erythroblast and normoblast. These samples showed the least batch effect. Mouse skin SHARE-seq data and DORC annotations were downloaded from GEO (GSE140203). The UMAP used for mouse skin was shared by the authors^1^. Pituitary multi-ome data were downloaded from GEO (GSE178454). The developing human cortex scRNA-seq was downloaded from GEO (GSE162170) and the corresponding multi-omic scATAC–seq was downloaded using the link https://github.com/GreenleafLab/brainchromatin/blob/main/links.txt. We used samples labeled hft_ctx_w21_dc2r2_r1 and hft_ctx_w21_dc2r2_r2 with the least batch effect. We subset the data to retain cells labeled as cycling progenitor, mGPC/OPC, SP, RG, nIPC/GluN1, GluN2, GluN3, GluN4 and GluN^57^. We downloaded the pancreas multi-ome dataset from the ENCODE portal (multi-omic series ENCSR233SQS) using the link https://www.encodeproject.org/multiomics-series/ENCSR233SQG/. PCHi-C data^14^ were downloaded from https://osf.io/u8tzp/. We used common and low-frequency variants (MAF ≥ 5) from the 1000 Genomes Project, phase 3 (ref. ^59^). The fine-mapped eQTLs for whole blood, pancreas and pituitary were downloaded from GTEx v8 (ref. ^15^). The fine-mapping was performed using CAVIAR^60^. The splicing QTLs (sQTLs) generated using LeafCutter^61^ were downloaded from GTEx v8. A *Q*-value cutoff of 0.05 was used to select the sQTLs. We also downloaded the conditionally independent eQTL from GTEx v8. UK Biobank GWAS data with fine-mapping using SuSIE^62^ and FINEMAP^63^ were downloaded from the Finucane Lab (https://www.finucanelab.org/data). Colocalization of GWAS and eQTL variants performed using the method coloc were downloaded from OpenTargets^24^ (https://ftp.ebi.ac.uk/pub/databases/opentargets/genetics/latest/v2d_coloc). LD blocks was downloaded from https://github.com/jmacdon/LDblocks_GRCh38/blob/master/data/pyrho_EUR_LD_blocks.bed. All SCARlink models and additional processed files required to generate the figures are available at https://figshare.com/s/9b9e89ff3150aebb6d7a (ref. ^64^).

## Code availability

SCARlink is available on GitHub at https://github.com/snehamitra/SCARlink/ (ref. ^65^). The notebooks to generate the figures are available within the same GitHub repository at https://github.com/snehamitra/SCARlink/tree/main/paper_figures (ref. ^65^).
