# Author Correction: Single-cell multi-ome regression models identify functional and disease-associated enhancers and enable chromatin potential analysis

**DOI:** 10.1038/s41588-024-01805-8

**Published:** 2024-05-23

**Authors:** Sneha Mitra, Rohan Malik, Wilfred Wong, Afsana Rahman, Alexander J. Hartemink, Yuri Pritykin, Kushal K. Dey, Christina S. Leslie

**Affiliations:** 1 Computational and Systems Biology Program, Memorial Sloan Kettering Cancer Center, New York City, NY USA; 2 Rye Country Day School, Rye, NY USA; 3 Tri-Institutional Training Program in Computational Biology and Medicine, New York City, NY USA; 4 Hunter College, City University of New York, New York City, NY USA; 5 Department of Computer Science, Duke University, Durham, NC USA; 6 Program in Computational Biology and Bioinformatics, Duke University, Durham, NC USA; 7 Center for Genomic and Computational Biology, Duke University, Durham, NC USA; 8 Department of Computer Science, Princeton University, Princeton, NJ USA; 9 Lewis-Sigler Institute for Integrative Genomics, Princeton University, Princeton, NJ USA

**Keywords:** Software, Population genetics, Epigenetics

Correction to: *Nature Genetics* 10.1038/s41588-024-01689-8, published online 21 March 2024

In the version of the article initially published, in the “Gene regression model” section in the Methods, the equation for the loss function was incorrect and should be:

$$\frac{1}{N}{\sum }_{i=1}^{N}\left({e}^{{X}_{i}{\boldsymbol{w}}+\epsilon }-{Y}_{i}\left({X}_{i}{\boldsymbol{w}}+\epsilon \right)\right)+\alpha {\left|\left|{\boldsymbol{w}}\right|\right|}_{2}^{2}$$

In the same section, the default proportion of held-out data in model evaluation has been corrected to 0.25 (one-quarter) rather than 0.2 (one-fifth) in the sentence “We left out one-fourth of the data for testing. The regularization parameter was selected using fourfold cross-validation on the remaining three-quarters of the cells.” These corrections have been made to the HTML and PDF versions of the article and do not change the results reported in the paper.

