# Supplementary material for: Single-cell multi-ome regression models identify functional and disease-associated enhancers and enable chromatin potential analysis
Source: Nat Genet. 2024 Mar 21;56(4):627–36. doi: 10.1038/s41588-024-01689-8 (PMC11018525; doi:10.1038/s41588-024-01689-8)
Supplement: Supplementary file 1 — Reporting Summary [file 41588_2024_1689_MOESM1_ESM.pdf]

nature portfolio

Corresponding author(s): Christina S. Leslie and Kushal K. Dey

Last updated by author(s): Jun 19, 2023

Reporting Summary

Nature Portfolio wishes to improve the reproducibility of the work that we publish. This form provides structure for consistency and transparency in reporting. For further information on Nature Portfolio policies, see our Editorial Policies and the Editorial Policy Checklist.

Statistics

For all statistical analyses, confirm that the following items are present in the figure legend, table legend, main text, or Methods section.

n/a	Confirmed	
☐	☒	The exact sample size (*n*) for each experimental group/condition, given as a discrete number and unit of measurement
☐	☒	A statement on whether measurements were taken from distinct samples or whether the same sample was measured repeatedly
☐	☒	The statistical test(s) used AND whether they are one- or two-sided<br>*Only common tests should be described solely by name; describe more complex techniques in the Methods section.*
☐	☒	A description of all covariates tested
☐	☒	A description of any assumptions or corrections, such as tests of normality and adjustment for multiple comparisons
☐	☒	A full description of the statistical parameters including central tendency (e.g. means) or other basic estimates (e.g. regression coefficient) AND variation (e.g. standard deviation) or associated estimates of uncertainty (e.g. confidence intervals)
☐	☒	For null hypothesis testing, the test statistic (e.g. *F*, *t*, *r*) with confidence intervals, effect sizes, degrees of freedom and *P* value noted<br>*Give P values as exact values whenever suitable.*
☒	☐	For Bayesian analysis, information on the choice of priors and Markov chain Monte Carlo settings
☒	☐	For hierarchical and complex designs, identification of the appropriate level for tests and full reporting of outcomes
☐	☒	Estimates of effect sizes (e.g. Cohen's *d*, Pearson's *r*), indicating how they were calculated

Our web collection on statistics for biologists contains articles on many of the points above.

Software and code

Policy information about availability of computer code

Data collection	N/A
Data analysis	Code availability: https://github.com/snehamitra/SCARlink<br><br>Software: Python (3.8.16), R (4.2.0)<br>Python packages: Tensorflow (2.11.0), shap (0.41.0), fa2 (0.3.5), scVelo0.2.5<br>R packages: Seurat (4.2.2), ArchR (1.0.2)

For manuscripts utilizing custom algorithms or software that are central to the research but not yet described in published literature, software must be made available to editors and reviewers. We strongly encourage code deposition in a community repository (e.g. GitHub). See the Nature Portfolio guidelines for submitting code & software for further information.

Data

Policy information about availability of data

All manuscripts must include a data availability statement. This statement should provide the following information, where applicable:

- Accession codes, unique identifiers, or web links for publicly available datasets
- A description of any restrictions on data availability
- For clinical datasets or third party data, please ensure that the statement adheres to our policy

Single-cell multiome data sets:
PBMC: 10X Genomics (https://www.10xgenomics.com/welcome?closeUrl=%2Fresources%2Fdatasets&lastTouchOfferName=PBMC%20from%20a%20Healthy%20Donor%20-%20Granulocytes%20Removed%20Through%20Cell%20Sorting%20%283k%29&lastTouchOfferType=Dataset&product=chromium&redirectUrl=%2Fresources%2Fdatasets%2Fpbmc-from-a-healthy-donor-granulocytes-removed-through-cell-sorting-3-k-1-standard-2-0-0)

BMMC: Part of the NeurIPS 2021 open problem. The data set was downloaded from GEO (GSE194122). We used BMMC samples labeled as site1_donor1, site1_donor2, site1_donor3, site2_donor1, site2_donor4, site2_donor5, site3_donor10, site3_donor6, site3_donor7, and site4_donor9.
Mouse skin SHARE-seq: Data and DORC annotations were downloaded from GEO (GSE104203). The UMAP used for mouse skin was shared by the authors (Ma et al., Cell 2020).

Pituitary: Data was downloaded from GEO (GSE178454).
Developing human cortex: scRNA-seq was downloaded from GEO (GSE162170) and the corresponding multiomic scATAC-seq was downloaded from links listed in https://github.com/GreenleafLab/brainchromatin/blob/main/links.txt. We used samples labeled hft_ctx_w21_dc2r2_r1 and hft_ctx_w21_dc2r2_r2.

Pancreas multiome: ENCODE portal (multiomic series ENCSR233SQS). Link: https://www.encodeproject.org/multiomics-series/ENCSR233SQG/

Mouse skin SHARE-seq and DORC annotations were downloaded from GEO (GSE140203). The UMAP used for mouse skin was shared by the authors.

The developing human cortex scRNA-seq was downloaded from GEO (GSE162170) and the corresponding multiomic scATAC-seq was downloaded from links listed in https://github.com/GreenleafLab/brainchromatin/blob/main/links.txt. We used samples labeled hft_ctx_w21_dc2r2_r1 and hft_ctx_w21_dc2r2_r2 with the least batch effect. We subset the data to retain cells labeled as cycling progenitor, mGPC/OPC, SP, RG, nIPC/GluN1, GluN2, GluN3, GluN4, GluN58.

PCHi-C data sets:

PCHi-C data was downloaded from https://osf.io/u8tzp/.

Variant data sets:

Common variants from the 1000 Genomes Project, phase 3: https://www.internationalgenome.org/category/phase-3/

fine-mapped eQTLs for whole blood, pancreas, and pituitary: GTEx v8 (https://storage.googleapis.com/gtex_analysis_v8/single_tissue_qtl_data/GTEx_v8_finemapping_CAVIAR.tar)

conditionally independent eQTL: GTEx v8 (https://storage.googleapis.com/gtex_analysis_v8/single_tissue_qtl_data/GTEx_Analysis_v8_eQTL_independent.tar).

splicing QTLs: GTEx v8 (https://storage.googleapis.com/adult-gtex/bulk-qtl/v8/single-tissue-cis-qtl/GTEx_Analysis_v8_sQTL.tar)

UK Biobank GWAS data with fine-mapping using SuSIE and FINEMAP: downloaded from the Finucane lab (https://www.finucanelab.org/data).

Colocalization results were downloaded from https://ftp.ebi.ac.uk/pub/databases/opentargets/genetics/latest/v2d_coloc.

Research involving human participants, their data, or biological material

Policy information about studies with human participants or human data. See also policy information about sex, gender (identity/presentation), and sexual orientation and race, ethnicity and racism.

Reporting on sex and gender	*Use the terms sex (biological attribute) and gender (shaped by social and cultural circumstances) carefully in order to avoid confusing both terms. Indicate if findings apply to only one sex or gender; describe whether sex and gender were considered in study design; whether sex and/or gender was determined based on self-reporting or assigned and methods used. Provide in the source data disaggregated sex and gender data, where this information has been collected, and if consent has been obtained for sharing of individual-level data; provide overall numbers in this Reporting Summary. Please state if this information has not been collected. Report sex- and gender-based analyses where performed, justify reasons for lack of sex- and gender-based analysis.*
Reporting on race, ethnicity, or other socially relevant groupings	*Please specify the socially constructed or socially relevant categorization variable(s) used in your manuscript and explain why they were used. Please note that such variables should not be used as proxies for other socially constructed/relevant variables (for example, race or ethnicity should not be used as a proxy for socioeconomic status). Provide clear definitions of the relevant terms used, how they were provided (by the participants/respondents, the researchers, or third parties), and the method(s) used to classify people into the different categories (e.g. self-report, census or administrative data, social media data, etc.) Please provide details about how you controlled for confounding variables in your analyses.*
Population characteristics	*Describe the covariate-relevant population characteristics of the human research participants (e.g. age, genotypic*

Population characteristics	*information, past and current diagnosis and treatment categories). If you filled out the behavioural & social sciences study design questions and have nothing to add here, write "See above."*
Recruitment	*Describe how participants were recruited. Outline any potential self-selection bias or other biases that may be present and how these are likely to impact results.*
Ethics oversight	*Identify the organization(s) that approved the study protocol.*

Note that full information on the approval of the study protocol must also be provided in the manuscript.

Field-specific reporting

Please select the one below that is the best fit for your research. If you are not sure, read the appropriate sections before making your selection.

☒ Life sciences ☐ Behavioural & social sciences ☐ Ecological, evolutionary & environmental sciences

For a reference copy of the document with all sections, see nature.com/documents/nr-reporting-summary-flat.pdf

Life sciences study design

All studies must disclose on these points even when the disclosure is negative.

Sample size	We used all the cells for each sample considered.
Data exclusions	By default we chose all the cells passing quality control across all samples in the published data sets unless we observed batch effect in a subset of samples. In such cases we retained samples showing least batch effect. We listed the the samples used for each study in the Data section.
Replication	N/A
Randomization	N/A
Blinding	N/A

Reporting for specific materials, systems and methods

We require information from authors about some types of materials, experimental systems and methods used in many studies. Here, indicate whether each material, system or method listed is relevant to your study. If you are not sure if a list item applies to your research, read the appropriate section before selecting a response.

Materials & experimental systems

n/a	Involved in the study	
☒	☐	Antibodies
☒	☐	Eukaryotic cell lines
☒	☐	Palaeontology and archaeology
☒	☐	Animals and other organisms
☒	☐	Clinical data
☒	☐	Dual use research of concern
☒	☐	Plants

Methods

n/a	Involved in the study	
☒	☐	ChIP-seq
☒	☐	Flow cytometry
☒	☐	MRI-based neuroimaging
